# ZSCAN21 mediates the pathogenic transcriptional induction of α-synuclein in cellular and animal models of Parkinson’s disease

**DOI:** 10.1038/s41419-025-07722-w

**Published:** 2025-05-16

**Authors:** Alina Kozoriz, Stéphan Mora, Maria-Alessandra Damiano, Iria Carballo-Carbajal, Annabelle Parent, Lorena Kumarasinghe, Miquel Vila, Iréna Lassot, Solange Desagher

**Affiliations:** 1 IGMM, University of Montpellier, CNRS, Montpellier, France; 2 Neurodegenerative Diseases Research Group, Vall d’Hebron Research Institute (VHIR)-Center for Networked Biomedical Research on Neurodegenerative Diseases (CIBERNED), Barcelona, Spain; 3 IRIM, University of Montpellier, CNRS, Montpellier, France; 4 Catalan Institution for Research and Advanced Studies (ICREA), Barcelona, Spain; 5 Institut de Neurociències (INc-UAB), Autonomous University of Barcelona (UAB), Barcelona, Spain

**Keywords:** Cell death in the nervous system, Cellular neuroscience, Parkinson's disease, Ubiquitin ligases, Transcription

## Abstract

The expression level of α-synuclein is thought to play a crucial role in the pathogenesis of Parkinson’s disease. However, little is known about the molecular mechanisms regulating the transcription of its gene, *SNCA*, particularly in the context of the disease. The transcription factor ZSCAN21 has been shown to act on *SNCA*, but whether ZSCAN21 is actually involved in the induction of *SNCA* transcription in Parkinson’s disease is unknown. To address this question, we used the MPTP mouse model and LUHMES-derived dopaminergic neuronal spheroids, subjected to Parkinson’s disease-related neurotoxins and mutations. We show that MPP^+^-treated spheroids recapitulate the main features of α-synuclein pathology and that MPP^+^-triggered transcriptional induction of *SNCA* is associated with ZSCAN21 stabilisation. Importantly, knock-down of ZSCAN21 prevents both the MPP^+^-triggered increase in α-synuclein mRNA and pre-mRNA levels in LUHMES-derived spheroids and the death of dopaminergic neurons in the *substantia nigra* of MPTP-treated mice. These effects are recapitulated by knockdown of TRIM17, a ZSCAN21 stabiliser which prevents its ubiquitination and degradation mediated by TRIM41. Moreover, reducing the interaction between ZSCAN21 and TRIM41, either by inserting Parkinson’s disease-associated mutations into the *TRIM41* gene or by preventing SUMOylation of ZSCAN21, results in both stabilisation of ZSCAN21 and induction of *SNCA*. Taken together, our data strongly suggest that ZSCAN21 is a crucial transcription factor for pathogenic α-synuclein expression and neurodegeneration in Parkinson’s disease, pointing to its regulators, TRIM17 and TRIM41, as original therapeutic targets for a neuroprotective treatment of Parkinson’s disease.

## Introduction

Parkinson’s disease (PD) is the second most common neurodegenerative disease after Alzheimer’s disease and is currently the fastest growing neurological disorder in the world [1]. This progressive age-related movement disorder results primarily from the massive and selective degeneration of dopaminergic (DA) neurons in the *substantia nigra pars compacta* (SNpc) [2]. Both environmental and genetic factors contribute to the onset of PD. More than 20 genes have been found to be mutated in rare inherited forms of the disease [3]. In addition, genome-wide association studies (GWAS) have provided convincing evidence that polymorphic variants in these genes also contribute to sporadic PD, which represents the majority of cases [3]. The first gene found to be mutated in familial PD is *SNCA* [4], which encodes α-synuclein, an abundant neuronal protein. Missense mutations or genomic multiplication of *SNCA* cause autosomal dominant familial PD, and GWAS have established variation at the *SNCA* locus as one of the most important genetic risk factors for sporadic PD [5, 6]. Although its normal functions remain elusive, α-synuclein clearly has a role in synaptic transmission [7]. The potential role of α-synuclein in PD pathogenesis is thought to be related to its propensity to aggregate, in particular when it accumulates or when it is mutated. Indeed, α-synuclein is the major protein component of Lewy bodies and Lewy neurites, cytoplasmic inclusions which are the main histopathological hallmark of the disease [8]. Pathological aggregates of α-synuclein can generate various cellular damages, such as mitochondrial dysfunction, oxidative stress and altered proteostasis [9]. In addition, it is well admitted that fibrillar forms of α-synuclein can spread in the brain, in a prion-like manner [10].

Importantly, accumulating data indicate that even a limited increase in the expression of wild-type (WT) α-synuclein can cause both familial and sporadic forms of PD [11–13]. First, duplications and triplications of the WT *SNCA* locus are sufficient to cause inherited forms of PD. In these families, the severity of the disease directly correlates with gene copy number, as well as with α-synuclein mRNA and protein levels [14]. Second, genetic variability at the *SNCA* locus associated with increased risk of PD has been implicated in the upregulation of α-synuclein [12]. For example, the expansion of Rep1 (a polymorphic dinucleotide repeat element located ≈10 kb upstream of the *SNCA* transcription start site) that confers elevated risk for sporadic PD, has been shown to increase *SNCA* transcription in cellular and animal models, as well as in the SN in humans [15]. Moreover, several single nucleotide polymorphisms (SNPs) at the *SNCA* locus, highly associated with increased risk for PD in GWAS, correlated with higher levels of α-synuclein in the cerebrospinal fluid of patients [16], or higher mRNA levels in the SN [17] or frontal cortex [18] of human post-mortem tissue, as well as in human induced pluripotent stem cells [19].

In addition to genetic factors, environmental factors may also contribute to PD by increasing α-synuclein expression. Measurements of *SNCA* mRNA levels in post-mortem tissues from sporadic cases produced contradictory results [13]. However, in the widely used mouse model of PD based on the systemic administration of the neurotoxin MPTP (1-methyl-4-phenyl-1,2,3,6-tetrahydropyridine), the mRNA level of α-synuclein is clearly increased in the SN [20, 21]. The toxic metabolite of MPTP, 1-methyl-4-phenylpyridinium (MPP^+^), is very similar to the herbicide paraquat. As pesticide exposure has been associated with an increased risk of PD [22], it is very possible that at least some environmental causes of PD are also related to elevated *SNCA* transcription.

Although α-synuclein expression is critical for PD pathogenesis, little is known about the molecular mechanisms regulating the transcription of its gene [12]. A few transcription factors have been shown to bind DNA regulatory elements in the *SNCA* gene, and to activate or repress its transcription, in rodent brain, rodent primary neurons and human neuronal cell lines [11, 12, 23]. However, the actual role of these transcription factors in PD is unknown. We and others have shown that ZSCAN21 binds to a regulatory element within *SNCA* intron 1 [20, 24, 25]. Overexpressing ZSCAN21 increased α-synuclein expression [20, 26], while silencing ZSCAN21 decreased it [20, 25, 27], in different neuronal cell cultures. ZSCAN21 belongs to the SCAN domain family of zinc-finger transcription factors [28]. Initially identified as a marker for granule neuron lineage in the central nervous system [29], it has been shown to be expressed throughout the brain [25]. Notably, ZSCAN21 is expressed in most of the DA neurons of the SN, both in humans and mice [30].

**Fig. 1:**
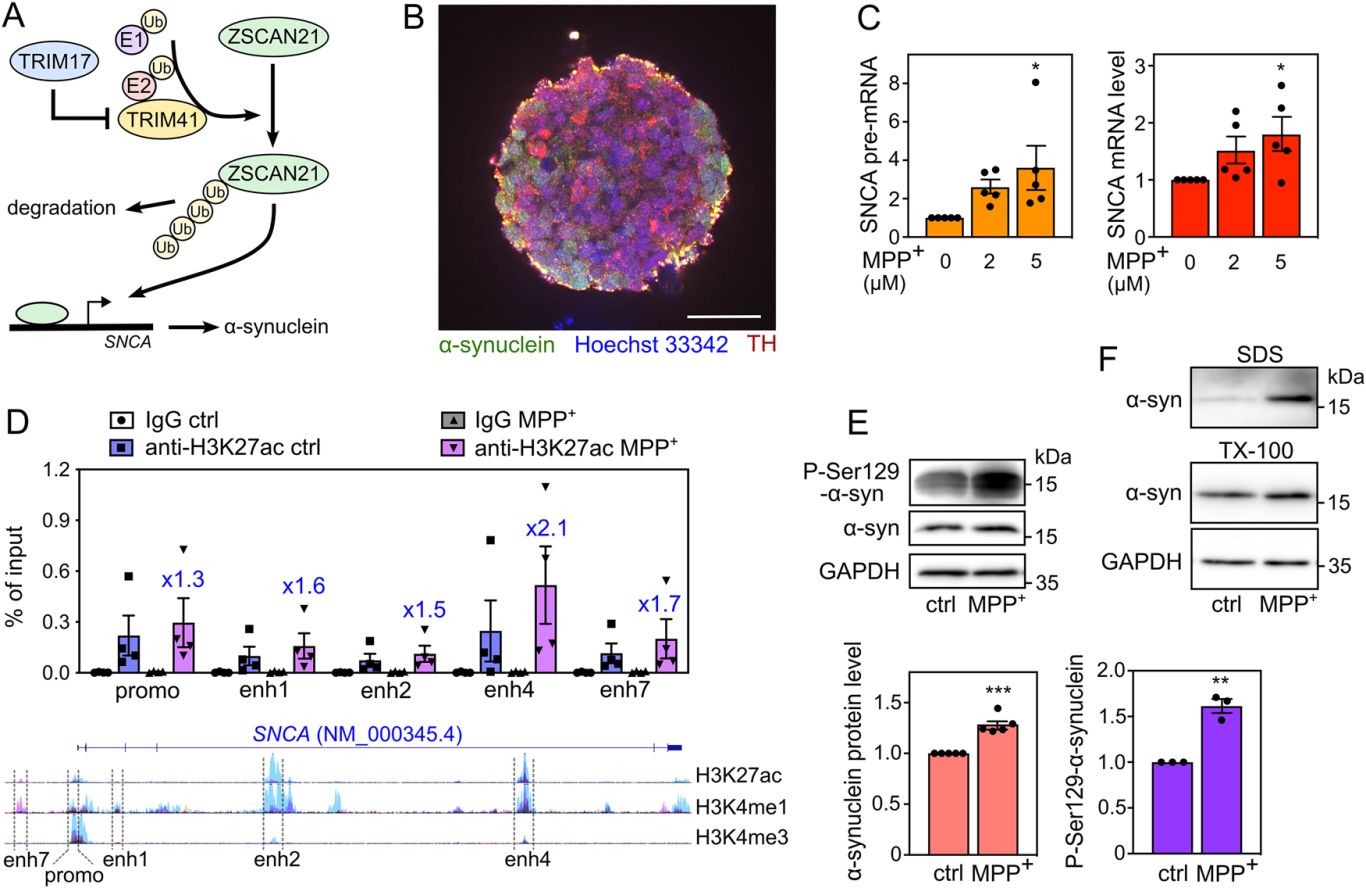
LUHMES-derived DA neuronal spheroids treated with MPP^+^ recapitulate α-synuclein upregulation and pathology. **A** Working hypothesis. Our previous results indicate that ZSCAN21 increases the transcription of the *SNCA* gene, TRIM41 is an E3 ubiquitin-ligase for ZSCAN21 that induces its proteasomal degradation, and TRIM17 inhibits TRIM41-mediated ubiquitination and degradation of ZSCAN21. **B** A spheroid of LUHMES-derived DA neurons after 7 days of differentiation. The spheroid was cleared, stained with antibodies against synuclein (green), TH (red) and Hoechst 33342 (blue), and analysed by confocal microscopy coupled with a spinning disk (Dragonfly). The scale bar is 50 μm. **C** After 6 days of differentiation, LUMHES spheroids were treated or not with 2 μM or 5 μM MPP^+^ for 24 h. Then, total RNA was extracted, and the pre-mRNA and mRNA levels of SNCA were estimated by quantitative RT-PCR, using *YWHAZ* and *TBP* as reference genes. The graph shows mean ± SEM and individual results of five independent experiments. ^*^Significantly different from non-treated cells (0) (one-way ANOVA followed by Dunnett’s multiple comparison test). **D** LUMHES spheroids were treated or not with 2 μM MPP^+^ for 24 h. ChIP assays were performed using an antibody against H3K27ac or rabbit IgG used as a negative control. Quantitative PCR was carried out on the immunoprecipitates and the input chromatin using primers specific for the promoter region and different enhancers. Data are expressed as the percentage of the input chromatin used for each immunoprecipitation and are the individual values and means ± SEM of four independent experiments. The ratios of enrichment in MPP^+^ vs control conditions with the H3K27ac antibody are indicated for each DNA sequence above the MPP^+^ columns. NB: the data are scattered because the enrichment rate is quite different from one ChIP experiment to another. However, in all experiments, a higher enrichment of all DNA sequences was seen in MPP^+^ vs control conditions, indicating a real reproducibility. Below the graph, a schematic representation of the human *SNCA* gene from the UCSC genome browser is shown. It depicts a single representative transcript (NCBI RefSeq NM_000345.4) and the epigenetic marks measured in ChIP-seq experiments on 7 cell lines from the ENCODE project. H3K27ac and H3K4me1 marks are often found near active regulatory elements, and H3K4me3 marks near promoters. The regulatory elements studied by ChIP are indicated. **E** LUMHES spheroids were treated or not with 2 μM MPP^+^ for 24 h. Then, total protein extracts were analysed by western blot using antibodies against α-synuclein, Ser129-phosphorylated α-synuclein and GAPDH. The intensity of the α-synuclein bands was quantified, normalised by the intensity of the GAPDH bands and expressed relative to the values obtained with non-treated cells. The graph shows mean ± SEM and individual points from three to five independent experiments. ^*^Significantly different from non-treated cells (unpaired *t*-test). **F** LUMHES spheroids were treated or not with 2 μM MPP^+^ for 29 h. Then, Triton-X100-soluble and SDS-soluble protein fractions were analysed by western blot using antibodies against α-synuclein and GAPDH. These data are representative of two independent experiments.

In a previous study, we showed that ZSCAN21 is regulated by two E3 ubiquitin-ligases from the TRIM family: TRIM41 which induces the ubiquitination and proteasomal degradation of ZSCAN21, and TRIM17 which stabilizes ZSCAN21 by inhibiting TRIM41 [20] (Fig. 1A). *ZSCAN21* was also recently identified as a novel candidate gene for PD. Indeed, we and others have found rare genetic variants of *ZSCAN21* in familial cases that co-segregate with the disease [20, 30]. These variants affect highly conserved amino acids at important positions, suggesting a potential involvement of ZSCAN21 in PD pathogenesis, possibly by increasing α-synuclein expression. However, as with the other transcription factors identified for *SNCA*, the role of ZSCAN21 in the pathological induction of α-synuclein in PD-associated conditions has not been explored.

Here, to determine whether endogenous ZSCAN21 is involved in the increase of *SNCA* transcription in PD-associated conditions, we developed a cellular model of human DA neurons that recapitulates α-synuclein pathology in response to the PD-related neurotoxin MPP^+^. Our data show that silencing ZSCAN21 or TRIM17 prevents the *SNCA* transcriptional induction triggered by MPP^+^ in this model, suggesting that ZSCAN21 and TRIM17 are indeed required for α-synuclein upregulation in pathological conditions. Consistently, silencing Zscan21 and Trim17 prevented DA neurodegeneration in the MPTP mouse model of PD. Moreover, a decrease in the TRIM41/ZSCAN21 interaction, either by a PD-related genetic variation of TRIM41 or by inhibition of ZSCAN21 SUMOylation, resulted in ZSCAN21 accumulation and increased mRNA level of α-synuclein. Therefore, our study strongly suggests that ZSCAN21 and its regulators TRIM17 and TRIM41 play a crucial role in the transcriptional induction of α-synuclein in PD.

## Results

### MPP^+^-induced transcription of *SNCA* in LUHMES-derived DA neuronal spheroids correlates with *TRIM17* induction and ZSCAN21 stabilisation

To recapitulate the pathological induction of the *SNCA* gene in a PD-relevant cellular model, we used the LUnd Human MESencephalic (LUHMES) cells. This cell line has been established by the conditional immortalisation (by tet-off-controlled v-myc) of neuronal precursors from the ventral midbrain of a human foetus [31]. These cells can be homogenously differentiated into post-mitotic DA neurons in one week, in the presence of tetracycline, cAMP and GDNF. Differentiated LUHMES cells show long neuritic extensions and electrical activity, and express all the characteristic markers of DA neurons [32]. To better model the in vivo physiology and gene expression in the human brain, we differentiated LUHMES cells in three dimensions (3D), by constant gyratory shaking as described by Smirnova et al. [33] (Fig. 1B). The resulting spheroids have been well characterised. Notably, they allow penetration by small molecules and a sufficient oxygen and nutrient supply for survival of the innermost cells [33]. Our immunostaining data indicate that differentiated LUHMES cells in the spheroids all express tyrosine hydroxylase, an important marker of DA neurons (Fig. 1B).

To mimic the pathological conditions of PD, we used MPP^+^, the toxic metabolite of MPTP, which reproduces the core neurological symptoms of PD in animal models. When 3D-differentiated LUHMES cells were incubated with 2 μM or 5 μM MPP^+^ for 24 h, the SNCA mRNA level was increased by a factor of 1.5–2 on average (Fig. 1C). As differentiated LUHMES cells have high steady-state levels of SNCA mRNA, we also measured the levels of SNCA pre-mRNA, by using primers in one exon and adjacent intron, to better detect the transcriptional induction of the *SNCA* gene. Indeed, the increase in SNCA pre-mRNA was also dose-dependent but stronger than the increase in SNCA mRNA levels (Fig. 1C).

To confirm the increase in *SNCA* transcription, we carried out chromatin immunoprecipitation (ChIP) assays using an antibody against histone H3-lysine 27 acetylation (H3K27ac), a histone mark generally associated with transcriptionally active regulatory elements. The enrichment in the immunoprecipitate compared to the input chromatin was measured by real-time PCR, using primers specific for the *SNCA* promoter region and different putative distal enhancer elements identified by the ENCODE project. As expected, the *SNCA* promoter was more active following MPP^+^ treatment, as well as all the enhancers we tested (Fig. 1D), confirming *SNCA* transcriptional induction. Interestingly, a putative distal enhancer element in intron 4, which we call “enhancer 4”, was more active in LUHMES spheroids and more activated following MPP^+^ treatment than other enhancers (Fig. 1D). The transcriptional induction of *SNCA* triggered by MPP^+^ resulted in a small but significant accumulation of α-synuclein protein (Fig. 1E). Importantly, the fraction of α-synuclein phosphorylated at Ser129 accumulated to a greater extent than total α-synuclein after MPP^+^ treatment (Fig. 1E). Consistently, sequential protein extraction into Triton-X100 (TX-100) and SDS fractions showed a higher amount of α-synuclein in Triton-insoluble fractions (SDS) after MPP^+^ treatment compared to the control condition (Fig. 1F), further suggesting that MPP^+^ induced α-synuclein pathology in LUHMES-derived DA neuronal spheroids.

**Fig. 2:**
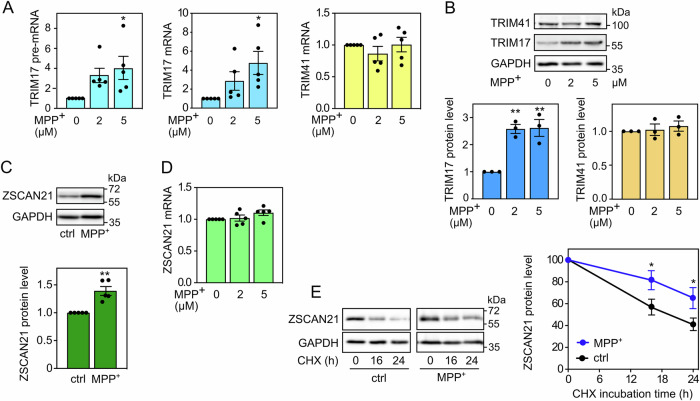
MPP^+^-induced *SNCA* transcription correlates with *TRIM17* induction and ZSCAN21 stabilisation. **A** After 6 days of differentiation, LUMHES spheroids were treated or not with 2 or 5 μM MPP^+^ for 24 h. Then, total RNA was extracted, and the pre-mRNA/mRNA levels of TRIM17 and the mRNA levels of TRIM41 were estimated by quantitative RT-PCR, using *YWHAZ* and *TBP* as reference genes. The graph shows mean ± SEM and individual results of five independent experiments. *: significantly different from non-treated cells (0) (one-way ANOVA followed by Dunnett’s multiple comparison test). **B** LUMHES spheroids were treated as in (**A**), and total protein extracts were analysed by western blot using antibodies against TRIM17, TRIM41 and GAPDH. The intensity of the TRIM protein bands was quantified, normalised by the intensity of the GAPDH bands and expressed relative to the values obtained with non-treated cells. The graph shows mean ± SEM and individual points from three independent experiments. ^*^Significantly different from non-treated cells (one-way ANOVA followed by Dunnett’s multiple comparison test). **C** LUMHES spheroids were treated or not with 2 μM MPP^+^ for 24 h. Then, total protein extracts were analysed by western blot using antibodies against ZSCAN21 and GAPDH. The results were analysed as in (**B**). The graph shows mean ± SEM and individual points from five independent experiments. *: significantly different from non-treated cells (unpaired *t*-test). **D** LUMHES spheroids were treated as in (**A**). Then, total RNA was extracted, and the mRNA level of ZSCAN21 was determined and analysed as in (**A**). **E** LUMHES spheroids were treated or not with 2 μM MPP^+^ for 24 h and with 40 μg/ml cycloheximide (CHX) for 0 h, 16 h or 24 h, as indicated. Total proteins were analysed by western blot using antibodies against ZSCAN21 and GAPDH. For each experiment, the amount of ZSCAN21 was normalised by the level of GAPDH in each condition and plotted against CHX incubation time. Data are the mean ± SEM of four independent experiments. ^*^Significantly different from non-treated cells at the indicated time point (two-way ANOVA followed by Sidak’s multiple comparison test).

In addition, the pre-mRNA, mRNA and protein levels of TRIM17 were increased in LUHMES spheroids treated with MPP^+^ compared to untreated spheroids (Fig. 2A, B). This is in line with previous reports showing that TRIM17 expression is increased following various cellular stresses [34, 35], notably in the SN of MPTP-treated mice [20]. In contrast, the mRNA and protein levels of TRIM41 did not change significantly (Fig. 2A, B). ZSCAN21 protein level was significantly increased by MPP^+^ treatment (Fig. 2C), although its mRNA level was not (Fig. 2D), indicating a post-translational mechanism. As TRIM17 can inhibit TRIM41-mediated ubiquitination and degradation of ZSCAN21 [20] (Fig. 1A), we estimated the half-life of ZSCAN21, after inhibition of protein synthesis using cycloheximide, in 3D-differentiated LUHMES cells treated with MPP^+^. Indeed, MPP^+^ treatment significantly stabilized ZSCAN21 (Fig. 2E), in line with the increased ZSCAN21 protein levels at the steady state level (Fig. 2C). Therefore, LUHMES-derived DA neuronal spheroids subjected to MPP^+^ support our working hypothesis: the cellular stress induced by MPP^+^ increases the expression of TRIM17; this, in turn, stabilizes ZSCAN21 by inhibiting TRIM41, which should result in increased *SNCA* transcription (Fig. 1A).

**Fig. 3:**
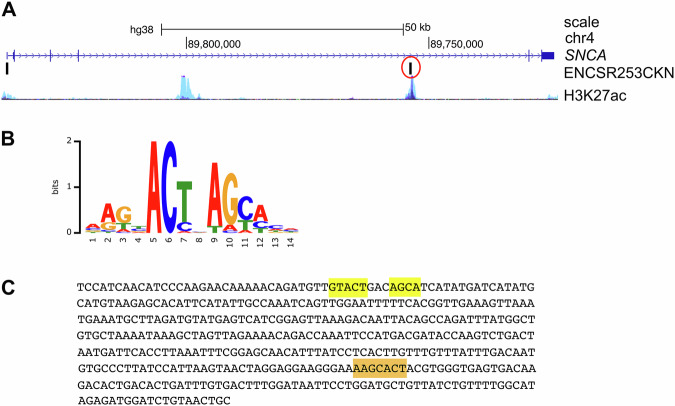
ZSCAN21 potentially binds to “enhancer 4” of *SNCA.* **A** Schematic representation of the human *SNCA* gene from the UCSC genome browser depicting a single representative transcript (NCBI RefSeq NM_000345.4), two peaks of eGFP-ZSCAN21 ChIP-seq in HEK293 cells from the ENCODE project (ENCSR253CKN) and the H3K27ac marks (often found near active regulatory elements) on seven cell lines from the ENCODE project. **B** Consensus binding sequence for ZSCAN21 from Factorbook (https://factorbook.org) determined by the MEME software using the eGFP-ZSCAN21 ChIP-seq data (ENCSR253CKN). **C** Sequence of the eGFP-ZSCAN21 ChIP-seq peak in “enhancer 4” circled in red in (**A**), with consensus binding sites for ZSCAN21 highlighted in orange and yellow.

ChIP-seq data from the ENCODE project performed on HEK293 cells expressing eGFP-ZSCAN21 (ENCSR253CKN) identified two peaks in the *SNCA* gene, one in the promoter region, and another one in “enhancer 4” (Fig. 3A). We downloaded the sequence of this second peak from the ENCODE portal [36] (https://www.encodeproject.org) and scanned it for the consensus binding site of ZSCAN21. The first consensus sequence, identified in 1996 by the SELEX method, was AGTAC, either as a single motif or two identical motifs in tandem separated by 2 or 7 nucleotides [29]. This consensus binding site was refined in 2017 as AAGBACTNAGCACH by using the eGFP-ZSCAN21 ChIP-seq data (ENCSR253 CKN) and the MEME software (https://factorbook.org) (Fig. 3B). We found the first complete part of this motif (AAGCACT) in the sequence immunoprecipitated with eGFP-ZSCAN21 (Fig. 3C, highlighted in orange), as well as the core consensus GTACT close to the second part of the motif AGCA (Fig. 3C, highlighted in yellow). This strongly suggests that ZSCAN21 can actually bind to “enhancer 4” in human *SNCA*. However, antibodies that specifically recognise endogenous human ZSCAN21 were not suitable for ChIP experiments, and we were unable to obtain convincing and reproducible chromatin enrichment with them.

### Knocking down TRIM17 and ZSCAN21 prevents induction of *SNCA* transcription in LUHMES cells and protects nigral DA neurons from MPTP in mice

**Fig. 4:**
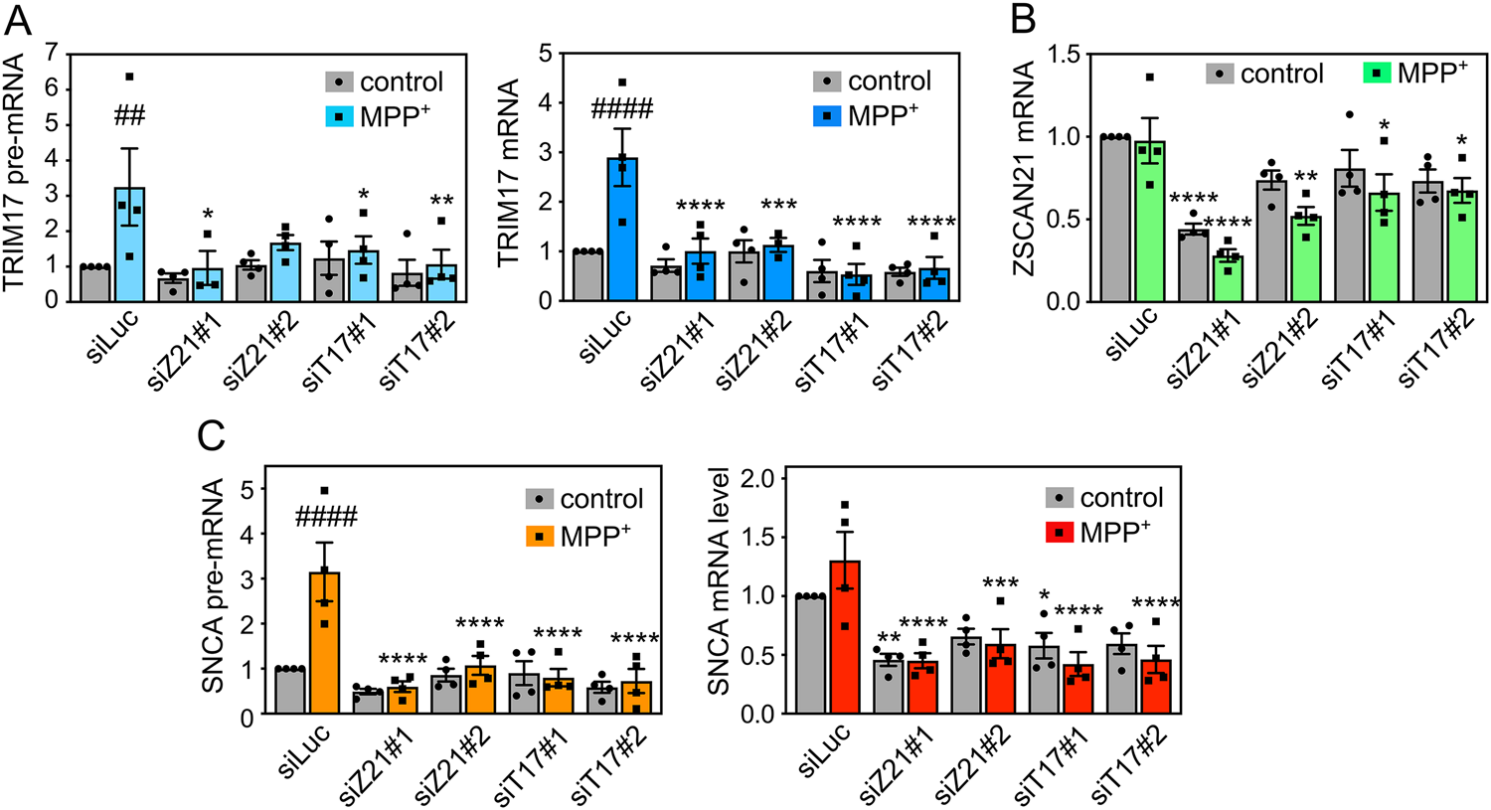
Knockdown of TRIM17 and ZSCAN21 prevents induction of *SNCA* transcription in LUHMES cells. LUHMES cells were transfected with a control siRNA against Luciferase (siLuc), two different siRNAs against TRIM17 (siT17#1 and siT17#2) or two different siRNAs against ZSCAN21 (siZ21#1 and siZ21#2) at the time of plating for 3D-differentiation. After 7 days of differentiation, LUMHES spheroids were treated or not with 2 μM MPP^+^ for 24 h and total RNA was extracted. The pre-mRNA and mRNA levels of TRIM17 (**A**), the mRNA level of ZSCAN21 (**B**) the pre-mRNA level and mRNA levels of SNCA (**C**) were estimated by quantitative RT-PCR, using *RSP16* and *HPRT1* as reference genes. The graphs show mean ± SEM and individual results of four independent experiments. ^*^Significantly different from siLuc in the same condition (control or MPP^+^, two-way ANOVA followed by Dunnett’s multiple comparison test). #: significantly different from cells treated with the same siRNA but not treated with MPP^+^ (two-way ANOVA followed by Sidak’s multiple comparison test).

To determine whether ZSCAN21 and its regulator TRIM17 are indeed involved in MPP^+^-induced *SNCA* transcription, we decreased ZSCAN21 and TRIM17 expression using specific siRNAs in 3D-differentiated LUHMES cells. The two different siRNAs against TRIM17 did not reduce the basal level of TRIM17 but significantly prevented the induction of TRIM17 expression following MPP^+^ treatment (Fig. 4A). One siRNA against ZSCAN21 (siRNA-ZSCAN21#1) significantly decreased ZSCAN21 expression in both control and MPP^+^ conditions, whereas the second one (siRNA-ZSCAN21#2) did it only in the MPP^+^ condition (Fig. 4B). The two siRNAs against TRIM17 and the two siRNAs against ZSCAN21 were also found to decrease the protein level of TRIM17 and ZSCAN21, respectively (Fig. S1). Strikingly, both knock-down of TRIM17 and ZSCAN21 almost completely abolished the increase of *SNCA* pre-mRNA level induced by MPP^+^ but did not affect its basal level (Fig. 4C). The MPP^+^-induced increase in *SNCA* mRNA levels was not statistically significant in this set of experiments, due to variability possibly resulting from the transfection of siRNAs which fragilizes the neurons. However, the trend was clearly an increase (Fig. 4C). Silencing of TRIM17 and ZSCAN21 not only prevented this induction but also tended to decrease the basal level of SNCA mRNA, in a significant way for siRNA-ZSCAN21#1 and siRNA-TRIM17#1 (Fig. 4C). Therefore, these data strongly suggest that the transcription factor ZSCAN21 and its stabilizer TRIM17 are involved in the transcriptional induction of *SNCA* in DA neurons following MPP^+^ treatment.

**Fig. 5:**
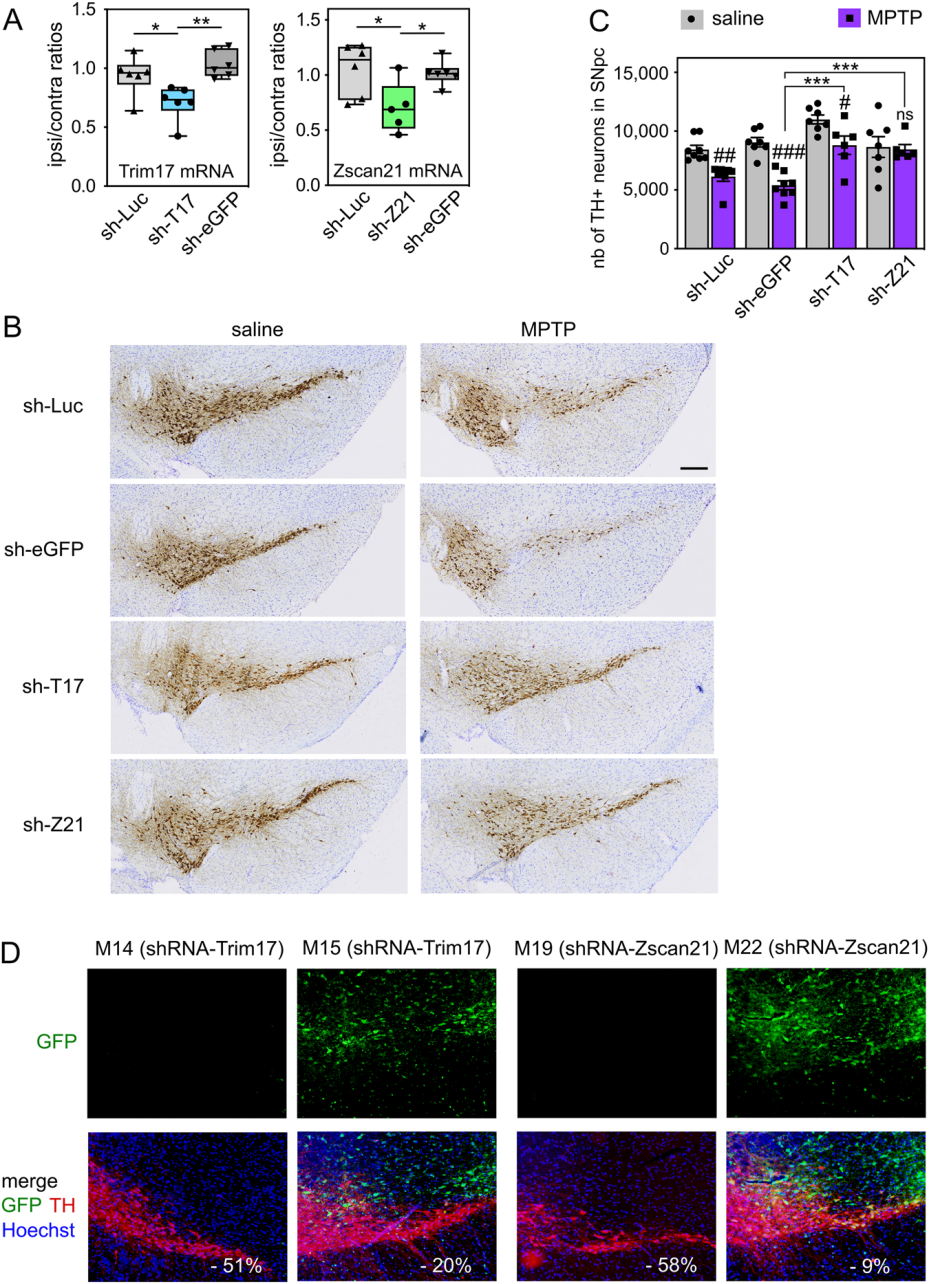
Knock-down of Trim17 and Zscan21 in SN protect DA neurons from MPTP-induced neurodegeneration in mice. **A** AAV vectors expressing control shRNAs (sh-Luc and sh-eGFP) or shRNAs against Trim17 (sh-T17) and Zscan21 (sh-Z21) were unilaterally injected in the midbrain of mice. Four weeks after injection, the mRNA levels of Trim17 and Zscan21 were measured both in the contralateral and ipsilateral midbrains of each mouse by RT-qPCR using *β2-microglobulin* and *Hmbs* as reference genes. Ipsilateral/contralateral mRNA ratios are presented as box plots, with minimum, maximum and median indicated. ^*^Significant difference (one-way ANOVA followed by Sidak’s multiple comparison test). **B** Four weeks after injection of AAV vectors as in A, mice were subjected to or not (saline) to subacute MPTP treatment and were sacrificed three weeks after the last MPTP injection. Brain sections were stained with anti-TH antibody by immunohistochemistry, and representative images of the ipsilateral SN in the different conditions are shown. The scale bar is 200 μm. **C** The total number of TH-positive neurons in the SNpc of mice was counted by stereology in the different conditions. ^#^Significantly different from control mice (saline) injected with the same AAV (two-way ANOVA followed by Sidak’s multiple comparison test); ^*^Significantly different from sh-eGFP in the MPTP condition (two-way ANOVA followed by Dunnett’s multiple comparison test). NB: differences are also significant when compared with sh-Luc; in saline conditions, only shT17 is different from sh-Luc or sh-eGFP. **D** Some brain sections from the same mice analysed in B were used for a double immunofluorescence staining of TH and GFP. Representative pictures of the ipsilateral SNpc of MPTP-treated mice are shown. For each mouse, the decrease of the total number of TH-positive neurons in the SNpc, compared to the mean of control mice (saline) injected with the same shRNA, is indicated in white. NB: in mice exhibiting a strong MPTP-induced neurodegeneration, GFP is not expressed in the SNpc, whereas in mice showing a strong neuroprotection, GFP expression is strong in the SNpc.

To examine whether these mechanisms may have an impact on DA neuronal death in PD conditions, we used the MPTP mouse model. We have previously shown that 4 days after subacute MPTP treatment, a significant increase of Trim17 and Zscan21 mRNA levels is concomitant with a peak of α-synuclein mRNA and is preceded by an important decrease in Trim41 expression [20]. These results suggest that the accumulation of Zscan21 protein that should result from these changes may contribute to the MPTP-induced increase in α-synuclein expression (Fig. 1A). In order to knock down Trim17 and Zscan21 in mice, we used recombinant adeno-associated viral vectors (AAV 2/1 serotype) co-expressing GFP and specific shRNAs. We selected shRNA sequences that effectively decrease the expression of Trim17 and Zscan21 in cultured mouse neurons [20]. In addition, we used two control shRNAs that do not alter the expression of our genes of interest and do not affect neuronal survival in vitro (shRNA-Luc and shRNA-eGFP) [20, 34]. The AAV vectors were unilaterally injected just above the right SN of mice, and their midbrains were analysed 4 weeks after the injection to estimate the level of gene silencing by RT-qPCR. The dissected tissues certainly contained a mix of transduced and non-transduced cells, thereby diluting the silencing effect. In spite of that, the ratios of Trim17 and Zscan21 mRNA levels between the ipsilateral and the contralateral midbrains, after injection of AAV vectors expressing shRNA-Trim17 or shRNA-Zscan21, were 0.709 ± 0.061 and 0.704 ± 0.102 (average ± SEM, *n* = 6 or 5), respectively. These ratios were significantly lower than the ipsilateral/contralateral ratios of Trim17 and Zscan21 mRNA levels, in the midbrains of mice injected with shRNA-Luc or shRNA-eGFP, which ranged from 0.940 to 1.053 on average (Fig. 5A). Therefore, the recombinant AAV vectors expressing specific shRNAs reduced the expression of endogenous Trim17 and Zscan21 in mice by at least 30%.

In a second series of experiments, mice were subjected to a subacute MPTP regimen four weeks after midbrain injection of the shRNA-expressing AAV vectors. Four weeks after the last MPTP injection, once DA neurodegeneration is stabilised in this model, ipsilateral brain sections were stained by immunohistochemistry for the DA marker tyrosine hydroxylase (TH), and the total number of DA neurons remaining in the SNpc was counted by stereology (Fig. 5B, C). As expected, MPTP significantly decreased the number of TH-positive neurons in the SNpc of mice injected with the control shRNAs: -27% with shRNA-Luc and −40% with shRNA-eGFP, compared to control mice injected with the same shRNAs but treated with saline buffer instead of MPTP (Fig. 5C). Importantly, this cell loss was lower with shRNA-Trim17 (−20%) and almost null with shRNA-Zscan21 (−2.3%) (Fig. 5C). Accordingly, the number of DA neurons remaining in the SNpc after MPTP treatment was significantly higher in mice injected with shRNA-Trim17 or shRNA-Zscan21 compared with mice injected with control shRNAs (Fig. 5C). Interestingly, in saline conditions, the number of TH-positive neurons in the SNpc of mice injected with shRNA-Trim17 was significantly higher compared with mice injected with control shRNAs (two-way ANOVA followed by Dunnett’s multiple comparisons test), suggesting that Trim17 knock-down may reduce basal neuronal death. To confirm that the neuronal death induced by MPTP is actually decreased by shRNA-Trim17 although it increases the number of TH-positive neurons in saline conditions, we performed one-sample *t*-tests with the values of each group of MPTP-treated mice, using the mean of the values from all groups of saline-treated mice as the hypothetical mean. The tests show that the number of TH-positive neurons is statistically different from the mean of all saline values for MPTP-treated mice injected with shRNA-Luc (*p* = 0.0003) and shRNA-eGFP (*p* < 0.0001) but is not significantly different for MPTP-treated mice injected with shRNA-Trim17 (*p* = 0.5874) and shRNA-Zscan21 (*p* = 0.1064).

In these experiments, we noted that some mice injected with the shRNA-Trim17 or shRNA-Zscan21-expressing AAVs showed an extensive neurodegeneration, whereas others showed complete neuroprotection. Therefore, we used a few brain sections of MPTP-treated mice for a double immunofluorescence staining of TH and GFP in the ipsilateral SNpc (Fig. 5D). Indeed, AAV vectors express both GFP and shRNAs, and the level of GFP expression is an indicator of shRNA expression and efficacy. As expected, the mice with a high neuroprotection (such as M15 and M22) showed an important GFP expression in the SNpc (Fig. 5D). In contrast, the few mice that exhibited extensive neurodegeneration (such as M14 and M19) showed almost no expression of GFP in the SNpc (Fig. 5D). GFP was detected in the ipsilateral midbrain of these mice but outside the SNpc. As the shRNAs were not well expressed in the SNpc of these mice, we excluded these mice from the analysis of stereological cell counts (Fig. 5C). Altogether, these data strongly suggest that knocking down Trim17 or Zscan21 in the SNpc protects DA from MPTP-induced neurodegeneration.

### Mutations of TRIM41 impair its interaction with ZSCAN21, resulting in ZSCAN21 stabilisation and increased α-synuclein expression

In a previous study, we identified two rare variants of the *TRIM41* gene in a cohort of patients with familial PD [20]. These variants were absent in healthy controls, and one of them was found to co-segregate with the disease. These two genetic variations affect two Arg residues (R534C and R536W amino acid substitutions) among a stretch of four Arg in the PRY-SPRY domain of TRIM41, in a region that is specific to TRIM41 and is absent in other PRY-SPRY domain-containing TRIM proteins. Interestingly, overexpression experiments in transformed cell lines showed that TRIM41 p.R534C interacts with ZSCAN21 with a lower affinity and increases α-synuclein expression compared with WT TRIM41 [20].

To further explore the hypothesis that these genetic variations may have contributed to the onset of PD in patients, we assessed their impact on ZSCAN21 and α-synuclein at the endogenous level. For this purpose, we used a genome editing technology, based on direct delivery of a Cas9-guide RNA ribonucleoprotein (RNP) complex previously assembled in vitro [37] together with a template for DNA repair, to introduce both mutations (R534C and R536W) in the *TRIM41* gene of LUHMES cells (Fig. S2). The donor DNA was an asymmetric 127 nt single-stranded oligonucleotide, complementary to the strand containing the PAM sequence, with 36 nt in 5’ and 91 nt in 3’ from the Cas9 cleavage site, to increase homology-directed repair [38]. The oligonucleotide contained the two point mutations to be introduced and was modified by two phosphorothioate molecules at each end, to increase DNA stability [39] (Fig. S2A). Following single-cell cloning, LUHMES clones were screened using a restriction test and positive clones were sequenced. Most clones were found to carry small deletions around the Cas9 cleavage site and were heterozygous. However, one clone harboured the two desired mutations on one allele and an in-frame 27 nt deletion on the other allele, resulting in the deletion of 9 amino acids, including the full stretch of four Arg (Fig. S2B). Sanger sequencing did not detect any other modification of genomic DNA around the targeted site. Therefore, we reasoned that this compound heterozygous clone, referred to as T41Mut hereafter, could be a useful tool to study the effect of genetic variations affecting the Arg stretch of TRIM41 in patients, as the two crucial Arg residues are lost on both alleles.

**Fig. 6:**
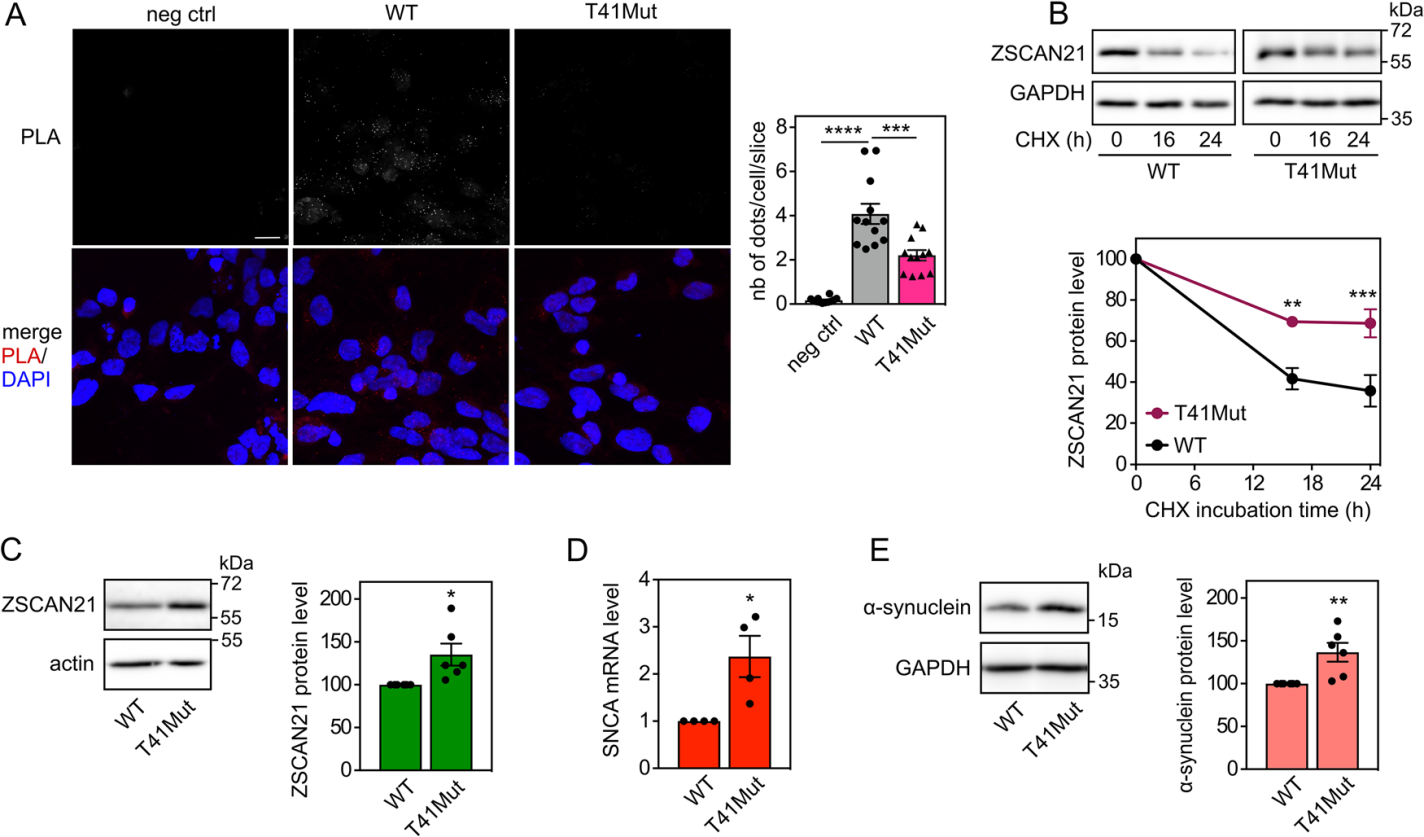
Mutations of TRIM41 impair its interaction with ZSCAN21, resulting in ZSCAN21 stabilization and increased α-synuclein expression. **A** 2D-differentiated LUHMES cells on a glass coverslip were fixed and subjected to in situ PLA using rabbit anti-TRIM41 and mouse anti-ZSCAN21 antibodies. Each bright spot (white or red in the merged picture) indicates that the two proteins are in close proximity. Negative control was obtained by omitting anti-ZSCAN21 antibody. Images were analysed by confocal microscopy. To better visualize the differences in PLA intensity, maximum intensity projection was applied to the z-stacks of images. Nuclear staining was performed using DAPI and visualised in merged pictures. The number of dots per cell was determined in one slice of each image using Fiji. The graph shows the mean ± SEM and individual data from 12 images per condition, including a total of more than 250 cells, from one experiment representative of two independent experiments. ^*^Significant difference (one-way ANOVA followed by Dunnett’s multiple comparison test). The scale bar is 10 μM. NB: because the size and intensity of the dots are reduced in T41Mut cells, they are barely visible in the figure, but they could be counted. **B** 3D-differentiated WT or T41Mut LUHMES cells were treated with 40 μg/ml cycloheximide (CHX) for 0 h, 16 h or 24 h. Total proteins were analysed by western blot using antibodies against ZSCAN21 and GAPDH. For each experiment, the amount of ZSCAN21 was normalised by the level of GAPDH in each condition and plotted against CHX incubation time. Data are the mean ± SEM of five independent experiments. ^*^Significantly different from WT cells at the indicated time point (two-way ANOVA followed by Sidak’s multiple comparison test). **C** WT or T41Mut LUHMES cells were differentiated into spheroids for 7 days. Then, total protein extracts were analysed by western blot using antibodies against ZSCAN21 and actin. The intensity of the ZSCAN21 bands was quantified, normalised by the intensity of actin and expressed relative to the values obtained with WT cells. The graph shows mean ± SEM and individual points from six independent experiments. ^*^Significantly different from WT cells (unpaired *t*-test). **D** WT or T41Mut LUHMES cells were differentiated as in (**C**). Then, the mRNA levels of SNCA were estimated by quantitative RT-PCR, using *GAPDH* and *GUSB* as reference genes. The graph shows mean ± SEM and individual results of four independent experiments. ^*^Significantly different from WT cells (unpaired *t*-test). **E** WT or T41Mut LUHMES cells were differentiated as in (**C**). Total protein extracts were analysed using antibodies against α-synuclein and GAPDH, and data were treated as in (**C**).

Using in situ proximity ligation assay (PLA) in 2D-differentiated LUHMES cells, we found that endogenous ZSCAN21 and TRIM41 proteins are in close proximity, both in the nucleus and the cytoplasm (Fig. 6A), in line with our previous data [20]. Interestingly, the PLA signal significantly decreased in LUHMES cells carrying TRIM41 mutations. Indeed, in T41Mut cells, not only was the number of dots per cell approximately halved, but the size and intensity of the dots were also dramatically reduced (Fig. 6A) compared to WT cells. Alteration of the Arg stretch of TRIM41 may prevent the formation of multiprotein complexes, which is a common feature of TRIM proteins [40], providing a possible explanation for this decrease in the size and intensity of PLA dots. Therefore, these data strongly suggest that the interaction between endogenous ZSCAN21 and TRIM41 proteins is impaired by mutation/deletion of the Arg stretch in the PRY-SPRY domain of TRIM41, confirming previous experiments with overexpressed proteins [20].

As TRIM41 is an E3 ubiquitin-ligase for ZSCAN21, we examined whether TRIM41 mutations could also impact the stability of ZSCAN21. Indeed, the half-life of endogenous ZSCAN21 (Fig. 6B) and the protein level of ZSCAN21 at the steady state level (Fig. 6C) were significantly increased in T41Mut cells compared to WT LUHMES cells. This accumulation of ZSCAN21 was associated with a significant increase in α-synuclein mRNA (Fig. 6D) and protein levels (Fig. 6E). Altogether, these data suggest that alterations in the Arg stretch of TRIM41 PRY-SPRY domain may favour PD by impairing the TRIM41/ZSCAN21 interaction, thereby stabilising ZSCAN21 and increasing α-synuclein expression.

### Inhibition of SUMOylation impairs TRIM41/ZSCAN21 interaction, resulting in ZSCAN21 accumulation and increased α-synuclein expression

**Fig. 7:**
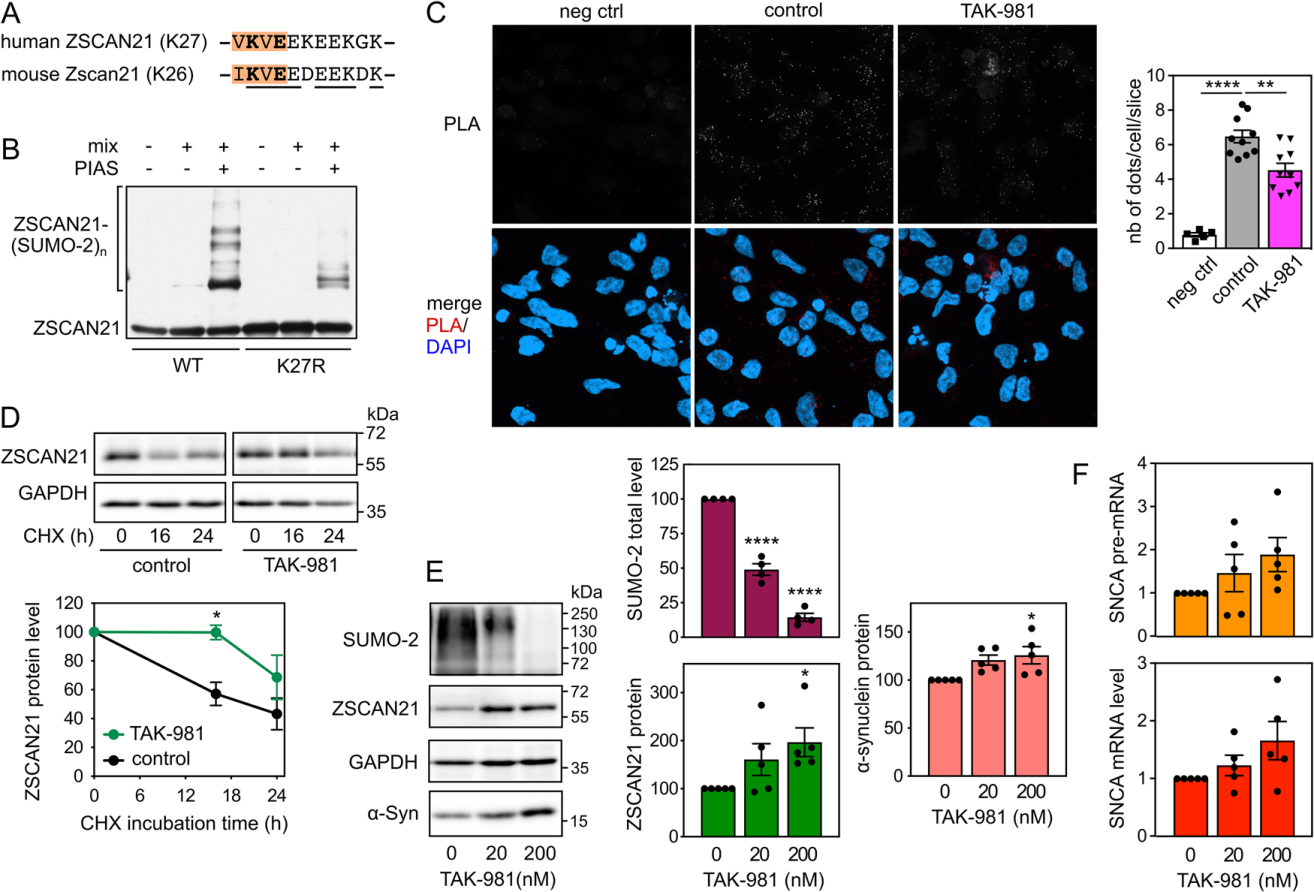
Inhibition of SUMOylation impairs the TRIM41/ZSCAN21 interaction, resulting in ZSCAN21 stabilisation and increased α-synuclein expression. **A** Negatively charged amino acid-dependent SUMOylation motif (NDSM) containing K27 of human ZSCAN21 and K26 of mouse Zscan21. The core consensus sequence (ΨKxE, where Ψ is a hydrophobic residue and x any amino acid) is in orange. The acceptor Lys residue and the determining Glu residue are in bold. Amino acids conserved between human and mouse are underlined. **B** In vitro translated 3 × Flag-ZSCAN21 (either the WT form or the K27R mutant) was incubated with in vitro SUMOylation reaction mix (containing SUMO-2, E1 and E2 enzymes), in the presence or the absence of the E3 enzyme PIASxα, as indicated, for 1 h at 37 °C. Poly-SUMOylated forms of ZSCAN21 were detected by immunoblotting using anti-Flag antibody. **C** 2D-differentiated LUHMES cells were treated with 200 nM TAK-981 for 24 h. Then, neurons were fixed and subjected to in situ PLA, and images were analysed as described in Fig. 6A. The graph shows the mean ± SEM of 10 images per condition, including a total of more than 220 cells (except for negative control), from one experiment representative of three independent experiments. ^*^Significant difference (one-way ANOVA followed by Dunnett’s multiple comparison test). **D** 3D-differentiated LUHMES cells were treated or not with 200 nM TAK-981 for 24 h and 40 μg/ml cycloheximide (CHX) for 0 h, 16 h or 24 h. Total protein extracts were analysed by western blot using antibodies against ZSCAN21 and GAPDH. For each experiment, the amount of ZSCAN21 was normalised by the level of GAPDH in each condition and plotted against CHX incubation time. Data are the mean ± SEM of four independent experiments. ^*^Significantly different from non-treated cells at the indicated time point (two-way ANOVA followed by Sidak’s multiple comparison test). **E** 3D-differentiated LUHMES cells were treated with 0 nM, 20 nM or 200 nM of TAK-981 for 24 h. Total protein extracts were analysed by western blot using antibodies against SUMO-2, ZSCAN21, α-synuclein and GAPDH. The intensity of the bands was quantified, normalized by the intensity of the GAPDH bands and expressed relative to the values obtained with non-treated cells. The graph shows mean ± SEM and individual points from five independent experiments. ^*^Significantly different from non-treated cells (one-way ANOVA followed by Dunnett’s multiple comparison test). **F** 3D-differentiated LUHMES cells were treated with 0 nM, 20 nM, or 200 nM of TAK-981 for 24 h. Then, total RNA was extracted, and the pre-mRNA and mRNA levels of SNCA were estimated by quantitative RT-PCR, using *B2M* and *RSP16* as reference genes. The graph shows mean ± SEM and individual results of five independent experiments. The statistical analysis does not show any significant difference.

To further confirm that the interaction between endogenous TRIM41 and ZSCAN21 proteins is important for α-synuclein transcriptional regulation, we tested another way of altering it. We showed in previous studies that some TRIM proteins preferentially interact with the SUMOylated forms of their partners [41, 42]. Therefore, we searched for a conserved consensus SUMOylation site in ZSCAN21. According to the web-based tool JASSA [43], K27 of human ZSCAN21 and K26 of mouse Zscan21 (Fig. 7A) are predicted to be the acceptor Lys residue of a negatively charged amino acid-dependent SUMOylation motif (NDSM) with a high confidence score. ZSCAN21 has actually been shown to be SUMOylated in cells by SUMO-2 at K27 in a comprehensive analysis of site-specific SUMO proteomics [44]. Using an in vitro SUMOylation assay, we confirmed that ZSCAN21 can be SUMOylated and that the K27R mutation strongly decreases its conjugation to SUMO-2 (Fig. 7B). Mutation of the determining Glu residue of the core SUMOylation site (ΨKxE) also strongly decreased the in vitro SUMOylation of ZSCAN21 by both SUMO-1 and SUMO-2 (Fig. S3).

We then tested the impact of inhibiting SUMOylation on the TRIM41/ZSCAN21 interaction using PLA. To this end, we used TAK-981, a selective inhibitor of SAE, the E1 SUMO-activating enzyme [45]. TAK-981 treatment did significantly decrease the PLA signal in LUHMES cells-derived DA neurons, suggesting a reduced interaction between endogenous TRIM41 and ZSCAN21 proteins following inhibition of SUMOylation (Fig. 7C). This was associated with a stabilization of endogenous ZSCAN21 (Fig. 7D). The dose-dependent and substantial decrease of global protein SUMOylation by TAK-981 was also associated with an increase in ZSCAN21 and α-synuclein protein levels (Fig. 7E). Although not statistically significant, SNCA pre-mRNA and mRNA levels also tended to increase after TAK-981 treatment (Fig. 7F). Taken together, these data suggest that inhibition of ZSCAN21 SUMOylation may favour PD by impairing the ZSCAN21/TRIM41 interaction, thereby promoting ZSCAN21 accumulation and increasing α-synuclein protein levels.

## Discussion

Although α-synuclein expression plays a central role in PD, and potentially in other neurodegenerative conditions, little is known about the mechanisms regulating the transcription of its gene. In particular, ZSCAN21 has been shown to regulate *SNCA* transcription in several independent studies [20, 24–27, 46]. However, its potential role as an inducer of α-synuclein expression in pathological conditions associated with PD has not been investigated.

Here, to address this question, we first developed a PD-relevant cellular model. Treatment of LUHMES-derived DA neuronal spheroids with micromolar concentrations of MPP^+^ increased *SNCA* pre-mRNA levels in a dose-dependent manner, indicating a transcriptional induction of the gene. This was confirmed by the H3K27ac ChIP-qPCR analysis of the *SNCA* gene, which showed that the promoter and some enhancers were more transcriptionally active after MPP^+^ treatment. Importantly, “enhancer 4” was particularly active and particularly activated following MPP^+^ treatment, compared to the other enhancers. This regulatory element in intron 4 has been recently described as a super enhancer, which is in contact with the *SNCA* promoter in SH-SY5Y cells and plays a predominant role in the regulation of the *SNCA* gene in the brain in vivo [47]. H3K27 has also been shown to be hyperacetylated at this enhancer in the brain of PD patients compared to healthy controls [48]. In addition, a PD-associated SNP located in this enhancer has been reported to increase α-synuclein expression in human neurons and in the brain of PD patients [19]. Therefore, “enhancer 4” appears to play a crucial role in the pathological activation of the *SNCA* gene. Its particular activation, following MPP^+^ treatment, in LUHMES spheroids, underlines the relevance of our cellular model. Moreover, in this model, the SNCA mRNA level was increased by a factor of 1.5–2, an increase similar to what is observed in MPTP-treated mice [20, 21] or in the brains of patients with a duplication or triplication of the *SNCA* locus [12]. This resulted in a significant increase of the α-synuclein protein and its phosphorylation at Ser129, a characteristic modification of disease-associated α-synuclein aggregates and Lewy bodies [49]. Protein extracts from Triton-insoluble fractions also exhibited more α-synuclein after MPP^+^ treatment. Although it is unlikely that aggregation occurs after only 24 h of MPP^+^ treatment, these data suggest that MPP^+^ initiates pathological α-synuclein modification in LUHMES-derived DA neuronal spheroids. Therefore, our cellular model recapitulates some of the main features of PD-associated α-synuclein pathology.

Using spheroids of LUHMES-derived DA neurons, we found a correlation between different events leading to ZSCAN21 accumulation on the one hand and increased α-synuclein expression on the other hand. First, MPP^+^ treatment induced the expression of TRIM17, which is known to favour the stabilisation and accumulation of ZSCAN21 by inhibiting its E3 ubiquitin-ligase TRIM41 [20]. Indeed, the increased expression of TRIM17 correlated with both increased ZSCAN21 half-life, ZSCAN21 accumulation and enhanced *SNCA* transcription in MPP^+^-treated LUHMES spheroids. Second, alteration of the native *TRIM41* gene of LUHMES cells in a region affected by PD-associated genetic variations impaired the interaction between endogenous TRIM41 and ZSCAN21 proteins. This resulted in both ZSCAN21 stabilisation and increased α-synuclein protein levels in LUHMES spheroids. Third, impairment of the TRIM41/ZSCAN21 interaction by inhibition of SUMOylation had the same effect. The correlation between ZSCAN21 accumulation and α-synuclein expression in these different cellular contexts suggests that ZSCAN21 activates *SNCA* transcription. Indeed, we and others have shown that ZSCAN21 overexpression increases α-synuclein mRNA levels [20, 26] and that ZSCAN21 binds to an important regulatory element within *SNCA* intron 1, in primary rat cortical neurons [25] and SH-SY5Y cells [20], using ChIP-qPCR. Moreover, ChIP-seq data from the ENCODE project show that ZSCAN21 also binds to a sequence in “enhancer 4” that contains two of its consensus binding sites. Since “enhancer 4” plays an important role in the pathological induction of *SNCA* transcription, these data suggest that ZSCAN21 contributes to this process. We directly tested this hypothesis by knocking down ZSCAN21 and its stabiliser TRIM17 in LUHMES cells. Following 3D-differentiation into DA neurons, this silencing of ZSCAN21 and TRIM17 prevented the increase in *SNCA* pre-mRNA level induced by MPP^+^ and tended to decrease the basal levels of *SNCA* mRNA. These results indicate that ZSCAN21 and TRIM17 are indeed necessary for the transcriptional induction of *SNCA* in our cellular model. Further investigation will determine whether this is also the case with other PD-associated stimuli.

Importantly, silencing Zscan21 and Trim17 in mouse SN also prevented DA neuronal loss following MPTP treatment, strongly suggesting that Zscan21 and Trim17 are also necessary for MPTP-induced neurodegeneration in mice. The neurotoxin MPTP has been widely used to mimic PD in animal models [50]. Its toxic metabolite MPP^+^ is transported by the dopamine transporter into DA neurons, where it blocks the complex I of the respiratory chain, thereby leading to the selective degeneration of DA neurons [50]. Therefore, although the MPTP model does not fully recapitulate PD, with a rapid degeneration in the absence of Lewy body pathology, it mimics well the complex I defects observed in PD patients [51] and more generally the mitochondrial dysfunction that plays a central role in PD pathogenesis [52]. Moreover, α-synuclein mRNA levels are increased in the SN of MPTP-treated mice [20, 21], and Snca knock-out mice are resistant to MPTP-induced neurodegeneration [53, 54], suggesting that α-synuclein upregulation does play a role in this DA neuronal loss. Therefore, on the basis of our results in MPP^+^-treated LUHMES spheroids, it is tempting to hypothesise that the protection of DA neurons from MPTP by the knockdown of Zscan21 and Trim17 may be due to the prevention of *Snca* transcriptional induction in vivo. However, we did not examine whether the increase in *Snca* mRNA levels in the SN of mice 4 days after MPTP treatment [20] was indeed prevented by silencing of Zscan21 and Trim17. Further investigation is required to address this question, which is a limitation of our present study.

Measurements of *SNCA* mRNA in post-mortem tissues from sporadic cases have been inconsistent [13], showing either increased [55] or decreased [56] α-synuclein expression, or no difference [17], in the SN of PD patients compared with healthy controls. These discrepancies might be due to the fact that 60-80% of DA neurons in the SN have already been lost in patients at the time of diagnosis [2], as well as to the cellular heterogeneity of brain samples analysed by bulk RNA sequencing. Indeed, an elegant study using laser-captured microdissection, clearly demonstrated elevated α-synuclein mRNA levels in individual, surviving DA neurons in the SNpc from idiopathic PD brains compared with control brains [57]. Although the question of *SNCA* induction in the brain of sporadic patients remains controversial, it is clear that early molecular mechanisms leading to DA neuronal death in PD are very difficult to assess from post-mortem samples. In mice, the peak of Snca mRNA level is reached 4 days after the last MPTP injection, and no increase is visible when neurodegeneration is established, 3 weeks after treatment. This underlies the difficulty of studying the dynamics of pathogenesis in human post-mortem samples and the need for experimental models.

Although we could detect a clear increase in pre-mRNA and mRNA levels of *SNCA* in MPP^+^-treated LUHMES-derived DA neuronal spheroids, previous transcriptomics studies using LUHMES cells differentiated in classical monolayers did not report any significant increase in *SNCA* expression following MPP^+^ treatment [58, 59]. This apparent discrepancy might be due to the more physiological neuronal microenvironment provided by spheroids compared to 2D (two-dimensional) cultures. Indeed, traditional monolayer cell cultures fail to recapitulate some crucial features of in vivo physiology, such as the tight packing of cells in a tissue structure, which has an important impact on cell maturation and gene expression [60, 61]. We also noticed that removing tetracycline from the medium after five days of differentiation increased MPP^+^-induced *SNCA* transcription. This is possibly related to the neuroprotective effect that tetracyclines have in the context of PD [62], in particular by acting on α-synuclein [63]. The 3D environment of our cellular model and the removal of tetracycline after differentiation probably account for the differences in *SNCA* gene regulation compared with 2D-differentiated LUHMES cells.

Our data strongly suggest that ZSCAN21 plays a crucial role in the transcriptional induction of α-synuclein in DA neurons in PD-associated conditions. However, a previous study conducted by the group of Leonidas Stefanis reported more complex effects, with shRNA-mediated knock-down of ZSCAN21 increasing α-synuclein expression in neuronal rat primary cultures, but decreasing it in rat differentiated neurospheres, and having no effect in early post-natal rat hippocampus in vivo [25]. These contradictory results might be due to differences in cell culture or developmental stage. It is important to note that none of these models were dopaminergic or human. More importantly, this study was performed in normal conditions without any disease-associated stimulus. Nevertheless, the results reported by the group of Stefanis in the hippocampus of adult rats indicate that AAV-mediated silencing of ZSCAN21 did decrease α-synuclein mRNA levels, although the difference was not statistically significant [25]. Our previous data using SH-SY5Y cells [20], which have some DA characteristics, and the present data using LUHMES-derived DA neurons both show a decrease in SNCA mRNA levels following ZSCAN21 knock-down, confirming results from Stefanis group in rat neurospheres and adult rat hippocampus [25].

The effect of ZSCAN21 knock-down on *SNCA* transcription in LUHMES spheroids was stronger following MPP^+^ treatment, suggesting that ZSCAN21 is a key transcription factor for the pathological induction of *SNCA* in DA neurons. This role is likely linked to its regulation by TRIM17. Indeed, we have previously shown that TRIM17 is expressed at very low levels in most conditions but is transcriptionally induced at a very high level following a cellular stress [64] such as trophic factor deprivation [34] or anti-cancer drugs [35]. Therefore, increased expression of TRIM17, leading to ZSCAN21 stabilisation through TRIM41 inhibition (Fig. 1A), may translate cellular stress, such as pesticide exposure, into increased α-synuclein expression, thereby contributing to PD pathogenesis. The inhibition of *SNCA* induction in MPP^+^-treated LUHMES spheroids and the protection of DA neurons in the SN of MPTP-treated mice by TRIM17 silencing are in line with this hypothesis. Variations in the *TRIM41* gene, resulting in decreased TRIM41/ZSCAN21 interaction [20], accumulation of ZSCAN21 and increased α-synuclein expression, also suggest that these genetic variations may have contributed to PD pathogenesis in the patients in whom they were identified. Altogether, our data point to ZSCAN21 and its regulators TRIM17 and TRIM41 as valuable therapeutic targets to prevent the pathological upregulation of α-synuclein, without affecting its basal level, in order to protect DA neurons and halt the progression of the disease.

## Materials and methods

### Materials

Culture media and TrypLE were from Thermo Fisher Scientific. Recombinant human basic Fibroblast Growth Factor (bFGF, #4114-TC-01M) and Glial cell line-Derived Neurotrophic Factor (GDNF, #202-GD-010) were from R&D systems. Paraformaldehyde 32% solution was from Electron Microscopy Sciences. Poly-L-ornithine, fibronectin, laminin, dibutyryl cAMP (dbcAMP), Paclitaxel, DAPI, protease inhibitor cocktail (P8340), TAK-981, mouse monoclonal anti-Flag antibody (clone M2, #F3165) and other chemicals were from Sigma-Aldrich. Mouse monoclonal anti-TH antibody (clone 2/40/15, #MAB5280) and mouse monoclonal anti-actin antibody (clone C4, #MAB1501) were from Merck Millipore. Rabbit monoclonal antibody against phosphor Ser129 α-synuclein (EP1536Y) and rabbit polyclonal anti-H3K27ac were from Abcam (#ab51253 and #ab4729, respectively). Mouse monoclonal anti-α-synuclein antibody was from BD Transduction Laboratories (#610786). Mouse monoclonal anti-ZSCAN21 antibody (clone OTI2E5, #TA506118) was from OriGene. Rabbit polyclonal anti-TRIM41 (#ARP34763) and anti-TRIM17 (#ARP34548) antibodies were from Aviva Systems Biology. Mouse monoclonal antibody against SUMO-2 (clone #8A2) was purified from hybridomas obtained from the Developmental Studies Hybridoma Bank. Rabbit polyclonal antibody against GAPDH was homemade. Fluorescent and horseradish peroxidase-conjugated goat anti-rabbit and anti-mouse secondary antibodies were from Thermo Fisher Scientific and Jackson ImmunoResearch Laboratories Inc., respectively.

### LUHMES cell culture and differentiation

LUHMES cells were a generous gift from Stefan Schildknecht from Marcel Leist’s laboratory in Konstanz, Germany. LUHMES cells were maintained in culture and differentiated in monolayers as previously described [31, 32] with a few modifications. Briefly, for proliferation of undifferentiated cells and 2D differentiation, LUHMES cells were maintained at 37 °C in a humidified 95% air and 5% CO_2_ atmosphere, in culture dishes previously coated with 50 μg/ml poly-L-ornithine and 1μg/ml fibronectin. When differentiated on glass coverslips, 10 μg/ml laminin was added to the coating solution. Proliferation medium comprised Advanced DMEM/F12 medium supplemented with N2 supplement, 2 mM GlutaMAX, 40 ng/ml bFGF and 100 IU/ml–100 μg/ml penicillin-streptomycin. Cells were passaged every 2–3 days: they were detached using TrypLE, diluted in Advanced DMEM/F12 medium, centrifuged at 300×*g* for 5 min and resuspended in proliferation medium. Cells were regularly tested for mycoplasma contamination.

For 2D differentiation, LUHMES cells were first seeded in proliferation medium: 2.6 million cells per 10 cm dish. The next day, they were switched to differentiation medium (Advanced DMEM/F12 medium supplemented with N2 supplement, 2 mM GlutaMAX, 1 mM dbcAMP, 1 μg/ml tetracycline, 2 ng/ml GDNF and 100 IU/ml–100 μg/ml penicillin-streptomycin). After 48 h in differentiation medium, cells were detached, counted and seeded at a density of 160,000 cells/cm^2^ in differentiation medium in suitable plates. After 5 days of differentiation, cells were washed with Advanced DMEM/F12 medium and incubated in tetracycline- and antibiotic-free differentiation medium for an additional 1–2 days before treatment and analysis.

Three-dimensional (3D) differentiation was performed as previously described [65]. LUHMES cells were detached using TrypLE, centrifuged and resuspended in differentiation medium containing 2 μg/ml instead of 1 μg/ml tetracycline. Cells in suspension were seeded in uncoated 6-well plates at 5.5 × 10^5^ cells/well in 2 ml differentiation medium and placed on an orbital shaker (STUART—SSM1) at 80 rpm in a humidified incubator at 37 °C with 10% CO_2_. After three days, the differentiation medium was partly changed and supplemented with 10 nM Paclitaxel, an anti-proliferation compound, for 48 h. On day 5 of differentiation, the resulting 300–400 μm spheroids were washed with Advanced DMEM-medium and incubated in tetracycline- and antibiotic-free differentiation medium for an additional 1–2 days before treatment and analysis.

### Transfection of LUHMES cells with siRNAs

LUHMES cells were transfected with siRNAs at the time they were switched to differentiation medium and placed on an orbital shaker for 3D differentiation. To reduce toxicity, the differentiation medium was free of tetracycline and other antibiotics at the time of transfection. Transfection using Lipofectamine RNAiMAX was performed according to the manufacturer’s instructions. For one well of a 6-well plate, 1.25 μl of transfection reagent was used with 12.5 pmoles of siRNA. The medium was changed after 4 h and replaced with full differentiation medium containing tetracycline and antibiotics.

### CRISPR/Cas9 editing

Synthetic RNAs and recombinant Cas9 protein (S.p. Cas9 nuclease 3NLS, #72643988) were provided by IDT (Alt-R™ CRISPR-Cas9 System). The RNP complex was assembled in vitro according to the manufacturer’s instructions. Briefly, equal volumes of 200 μM solutions of synthetic tracrRNA and crRNA were mixed, heated at 95 °C for 5 min and left to cool to room temperature on the bench to form the guide RNA. The custom crRNA targeted the PAM site nearest the sequence to be modified. The RNP complex was formed by mixing 1.2 μM of this guide RNA and 1 μM of purified Cas9 enzyme in sterile phosphate-buffered saline (PBS) and by incubating the mix for 15 min at room temperature. Meanwhile, proliferating LUHMES cells were detached in Versene solution (Thermo Fisher Scientific). Two million cells were centrifuged and resuspended in 90 μl of solution from the AMAXA Basic Nucleofector Kit for Primary Neurons (Lonza #VPI-1003). Ten microlitres of the CRISPR/Cas9 RNP complex and 2 μl of a 100 μM solution of the DNA template were added to the cells and mixed by gentle pipetting. The resulting cell suspension was transferred into an AMAXA cuvette, and cells were transfected using programme G013 of the AMAXA nucleofector device II. Immediately after nucleofection, 900 μl RPMI 1640 containing 10% FCS was added to the cuvette and cells were seeded in a 5 cm dish already containing 5 ml proliferation medium without antibiotics. Four to 8 h after transfection, the medium was replaced with complete proliferation medium.

Two days after transfection, cells were resuspended and sorted using flow cytometry (FACS Aria Becton-Dickinson) in order to seed one cell per well, in 96-well plates containing half volume of fresh proliferation medium and half volume of proliferation medium previously conditioned by growing proliferating LUHMES cells and filtered. Starting from one week after cell sorting, plates were checked every day to detect clones. Before reaching confluency, the clones were trypsinised and transferred into 24-well plates containing half conditioned medium and then to 6-well plates, and finally 5 cm dishes. During the transfer to 6 well plates, cells were counted and 90,000 cells were resuspended in 30 μl of QuickExtract™ DNA extraction solution (LGC Biosearch technologies SS000035-D1), homogenized by thorough vortexing for 15 s, incubated at 65 °C for 6 min, again homogenized by vortexing for 15 s, incubated at 98 °C for 2 min, and finally left to cool to room temperature. One to 5 μl of the resulting solution of genomic DNA was used in a PCR reaction to amplify a 773 nt sequence around the Cas9 cleavage site. The different clones were first screened by a restriction test. Indeed, desired mutations or small insertions/deletions around the Cas9 cut site disrupt a Nae1 restriction site, resulting in the appearance of a higher band after digestion. The PCR fragment of positive clones was further analysed by Sanger sequencing, whereas negative clones were discarded. To sequence the two alleles of the T41Mut clone separately, the PCR fragment was cloned into a plasmid, which was used to transform bacteria. Several DNA minipreps from individual colonies were sequenced.

### Immunofluorescence staining of LUHMES spheroids

LUHMES spheroids were immunostained as previously described [65, 66], with some modifications. Briefly, spheroids were fixed in 4% paraformaldehyde for 30 min at room temperature and washed three times with wash solution 1 (1% bovine serum albumin (BSA) in PBS). Fixed spheroids were permeabilised and cleared with optical clearing solution Scale S (20% Sorbitol, 10% glycerol, 4 M urea, 0.2% Triton-X100, in milli-Q water) for 48 h at 4 °C on a shaker. Cleared spheroids were again washed three times with wash solution 1 and then incubated in blocking solution (10% normal goat serum (NGS), 1% BSA and 0.15% saponin in PBS) for 1 h at room temperature. Blocking solution was removed, and spheroids were incubated with primary antibodies against the proteins of interest diluted in blocking solution, for 48 h at 4 °C on a shaker. Spheroids were then washed for 15 min three times with wash solution 2 (1% BSA and 0.15% saponin in PBS). Corresponding secondary antibodies coupled to fluorophores were diluted 1:1000 in blocking solution and incubated with spheroids for 48 h at 4 °C on a shaker. Spheroids were then washed for 15 min three times with wash solution 2, incubated with 1 μg/ml Hoechst 33342 in PBS for 1 h at room temperature on a shaker, washed twice with wash solution 1 and once with PBS. Spheroids were placed in Mowiol (polyvinyl alcohol 4–88, Fluka) in 8-well glass-bottom chambers (Ibidi) and analysed by confocal microscopy coupled with a spinning disk (Dragonfly Andor). Optical sectioning was performed by acquiring z-stacks at 2 μm intervals. Images were processed with Fiji.

### RNA extraction and quantitative RT-PCR

Total RNA was extracted using the RNeasy mini kit (Qiagen) and treated with DNase I from the DNA-free™ kit (Thermo Fisher Scientific) according to the manufacturer's instructions. RNA was used to perform a two-step reverse-transcription polymerase chain reaction (RT-PCR). In brief, 0.5–2 μg of total RNA was reverse-transcribed using 200 U reverse transcriptase Superscript II or Superscript IV for TRIM17 (Thermo Fisher Scientific) in the presence of 2.5 μM N6 random primers and 0.5 mM dNTP. The equivalent of 6 ng of resulting cDNA was used as a template for real-time PCR using a CFX Opus 384 real-time PCR system (BioRad) with a home-made SYBR Green PCR master mix [67]. PCR reactions were performed in 10 μl in the presence of 200 nM primers. Thermal cycling parameters were 5 min at 95 °C, followed by 40 cycles of 95 °C for 20 s, 64 °C for 20 s and 72 °C for 20 s, and a melt curve from 64 °C to 95 °C with 0.5 °C increments. Data were analysed and relative amounts of specifically amplified cDNA were calculated with CFX Maestro software (BioRad), using two different reference genes. For each type of experiment, the two most appropriate reference genes were determined by the geNorm software [68] from ten house-keeping genes (*B2M, GAPDH, GUSB, HMBS, HPRT1, RPL31, RSP16, SDHA, TBP, YWHAZ*).

### Protein extraction and western blot analysis

Cells were harvested in RIPA buffer (20 mM Tris-HCl [pH 7.5], 100 mM NaCl, 2.5 mM EDTA, 1% sodium deoxycholate, 1% Triton X-100, 1% sodium dodecyl sulfate), supplemented with phosphatase inhibitors (10 mM NaF, 5 mM sodium pyrophosphate, 25 mM β-glycerophosphate), protease inhibitor cocktail and 20 μM MG-132. Cells were lysed by thorough vortexing and sonication using the Bioruptor pico (Diagenode) with 20 cycles 30 s on, 30 s off. Cell debris was removed by centrifugation at 16.000×*g* for 5 min at 4 °C, and the protein concentration of the resulting supernatant was estimated using the BCA protein assay kit (Thermo Fisher Scientific) with BSA as the standard. Total lysates were diluted in 3 × Laemmli sample buffer and incubated at 95 °C for 5 min. Proteins were separated by 12% or 15% SDS–PAGE and transferred to Immobilon-P PVDF membrane (Millipore). Blocking and probing with antibodies were performed as previously described [20]. Visualisation of immunoreactive proteins was performed using horseradish peroxidase-linked secondary antibodies and Covalight enhanced chemiluminescent substrate (Covalab, Bron, France) or Immobilon® Western (Millipore). Membranes were revealed using Amersham Imager 680 (GE Healthcare). When necessary, membranes were stripped using Restore^TM^ Western Blot Stripping Buffer (Thermo Fisher Scientific) and re-probed with additional antibodies. Fiji software was used for optical density quantitation of Western blots.

### Sequential protein extraction into Triton X-100 and SDS fractions

Sequential protein extraction was performed as described in ref [69] with slight modifications. Briefly, LUHMES-derived spheroids from one well were collected by centrifugation, washed once with cold PBS and homogenized in 200 μl of Triton X-100 lysis buffer (50 mM Tris-HCl [pH 7.5], 150 mM NaCl, 5 mM EDTA [pH 8], 1% Triton X-100), supplemented with phosphatase inhibitors (10 mM NaF, 5 mM sodium pyrophosphate, 25 mM β-glycerophosphate), protease inhibitor cocktail and 20 μM MG-132. Lysates were incubated on ice for 30 min, sonicated using the Bioruptor pico (Diagenode) with 20 cycles 30 s on, 30 s off at 4 °C, and ultracentrifuged in polyallomer tubes (Beckman Coulter #357448) at 100,000×*g* for 30 min at 4 °C. The supernatant, corresponding to the Triton X-100 soluble fraction, was transferred into a tube, an aliquot was kept to measure protein content, and the rest was combined with 3× Laemmli sample buffer. The pellet resulting from the ultracentrifugation was washed with 200 μl of Triton X-100 lysis buffer, vortexed and ultracentrifuged again at 100,000×*g* for 30 min at 4 °C. The supernatant was discarded, and the pellet was resuspended in 50 μl of SDS buffer (50 mM Tris-HCl [pH 7.5], 150 mM NaCl, 5 mM EDTA [pH 8], 2% sodium dodecyl sulfate), supplemented with phosphatase inhibitors, protease inhibitor cocktail and 20 μM MG-132, to provide the SDS fraction. The pellet was completely dispersed by sonication as described above and diluted in 3× Laemmli sample buffer. Protein content was determined in the Triton X-100 fraction using the BCA protein assay kit. For each condition, 15 μg of proteins from the Triton X-100 extract and the same volume of SDS extract were separated by 15% agarose SDS-PAGE after adding fresh DTT and incubating the samples at 95 °C for 5 min. Proteins were transferred to PVDF membrane using the Trans-Blot Turbo transfer system (Bio-Rad). The membrane was incubated with 4% paraformaldehyde and 0.01% glutaraldehyde in PBS for 30 min at room temperature before blocking and addition of anti-α-synuclein antibody.

### ChIP

Spheroids were dissociated by incubation with TrypLE at 37 °C for 2 min and pipetting, fixed by the addition of formaldehyde to a final concentration of 1% and trituration/incubation for 20 min at 37 °C, before quenching by the addition of an equal volume of glycine 2.5 M for 5 min at room temperature. Cells were washed with PBS and incubated successively in wash buffer 1 (10 mM HEPES [pH 7.3], 0.25% Triton X-100, 10 mM EDTA, 0.5 mM EGTA, protease inhibitor cocktail) and in wash buffer 2 (10 mM HEPES [pH 7.3], 1 mM EDTA, 0.5 mM EGTA, 200 mM NaCl, protease inhibitor cocktail) for 10 min on ice. The cells were then incubated in lysis buffer (50 mM Tris-HCl [pH 7.5], 10 mM EDTA, 1% SDS, protease inhibitor cocktail). Each 120 μl aliquot of lysate was sonicated using the Minichiller 300 ultrasonicator (Diagenode) with two cycles (30 s on/30 s off). Lysates were centrifuged twice at 16.000 × *g* for 5 min at 4 °C. The DNA content of the resulting supernatants was measured, and ∼100 μg of chromatin was used for each immunoprecipitation. Samples were diluted 1:10 in ChIP dilution buffer (16.7 mM Tris-HCl [pH 8], 1.2 mM EDTA, 1.1% Triton X-100, 0.01% SDS, 167 mM NaCl, protease inhibitor cocktail) and were rotated overnight at 4 °C in the presence of anti-H3H27ac antibody or irrelevant mouse IgG together with protein G-agarose beads. The beads were then recovered by centrifugation at 1.500×*g* for 5 min at 4 °C and washed successively for 10 min at 4 °C with RIPA buffer (50 mM Tris-HCl [pH 8], 0.1% SDS, 0.5% sodium deoxycholate, 1% NP-40, 150 mM NaCl), high salt buffer (50 mM Tris-HCl [pH 8], 0.1% SDS, 1% NP-40, 500 mM NaCl), LiCl buffer (50 mM Tris-HCl [pH 8], 0.5% sodium deoxycholate, 1% NP-40, 250 mM LiCl) and twice with TE buffer (10 mM Tris-HCl [pH 8], 1 mM EDTA). The immune complexes were eluted by incubating the beads twice with 200 μl elution buffer (2% SDS, 100 mM NaHCO_3_, 1 mM DTT) for 15 min at room temperature and centrifuging them at 1.500×*g* for 5 min at 4 °C. The supernatants were collected, supplemented with 16 μl NaCl 5 M and incubated overnight at 65 °C to reverse protein-DNA cross-links. An equivalent quantity of input chromatin was treated the same way. RNA and proteins were removed from the samples by the addition of 8 μl EDTA 0.5 M, 16 μl Tris-HCl (pH 6.5) 1 M, 4 μl proteinase K (10 mg/ml), and 3.3 μl RNAse (24 mg/ml) and incubation for 1 h at 45 °C. Purified DNA from ChIP samples and input chromatin were then purified using the QIAquick PCR purification kit (Qiagen). Purified DNA was precipitated with NaHCO_3_ and ethanol, washed with 70% ethanol, dissolved in water and subjected to quantitative PCR. The amount of DNA precipitated by the antibodies was expressed as the percentage of the amount of input chromatin used for each immunoprecipitation.

### Animals

Eight- to 12-week-old male C57BL/6Ncrl male mice were used for all animal experiments. All the experimental and surgical procedures were conducted in accordance with the current legislation (see Ethics statement below). Sample size (eight animals per group) was chosen according to previous experience in the lab, and animals were allocated to different treatment groups by randomisation.

### Production and injection of AAV vectors

Recombinant AAV vectors (serotype 2/1) were produced by the Viral Vector Production Unit (Unitat de Producción de Vectors, UPV) of the Autonomous University of Barcelona (UAB, Spain). The shRNA sequences were cloned into pAAV-H1-RSV-GFP plasmid. This plasmid expresses shRNAs under the H1 RNA polymerase III promoter and eGFP under the RSV promoter. Briefly, recombinant AAV vectors were produced by triple transfection of HEK293 cells with pAAV, RepCap1 and pHelper using polyethylenimine (PEI; branched, MW 25,000; Sigma). The AAV particles were purified by iodixanol gradient as previously described [70] and were titrated following a method based on the quantitation of encapsidated DNA with the fluorescent dye PicoGreen® [71] and ranged from 7 to 11.10^12^ genome copies (gc)/ml.

Injections were performed with mice placed in a stereotaxic frame, under general anaesthesia using isoflurane. Vector solutions were injected using a 10 μl Hamilton syringe fitted with a glass capillary. One microliter of viral suspension was unilaterally injected into the area just above the right SNpc of mice (−2.9 mm antero-posterior, −1.3 mm lateral, and −4.2 mm dorso-ventral below the dural surface, relative to bregma) at a flow rate of 0.4 μl/min. The needle was left in place for an additional 4 min period before it was slowly retracted. Four weeks following AAV vector injection, mice were either euthanised to estimate the efficiency of shRNAs or treated with saline vehicle or MPTP, as indicated. For measuring the mRNA levels of Trim17 and Zscan21, mice were euthanised by cervical dislocation, the brain quickly removed, and the midbrain carefully dissected and snap-frozen on dry ice. Isolation of RNA from midbrain was performed using the RNeasy Mini Kit (Qiagen,) following the manufacturer’s instructions. Quantitative RT-PCR was performed with 300 ng of RNA as described above.

### Subacute MPTP intoxication, immunohistochemistry and immunofluorescence on midbrain sections, stereological counting

Mice received a single intraperitoneal injection of 1-methyl-4-phenyl-1,2,3,6-tetrahydropyridine (MPTP)-HCl per day (30 mg/kg/day of free base; Sigma-Aldrich, #M0896) for 5 consecutive days. Control mice received saline injections instead. *N* = 6–8 animals/group. Mice were euthanised 25 days after the last MPTP injection. Mice were deeply anaesthetised with an intraperitoneal injection of sodium pentobarbital (50 mg/kg) and then perfused through the left ventricle with physiological saline (0.9% NaCl) for 3 min, followed by 4% ice-cold paraformaldehyde diluted in 0.2 M phosphate buffer for 8 min, with a 9 ml/min flow rate. Brains were removed, post-fixed with 4% paraformaldehyde for 24 h at 4 °C, cryoprotected with 30% sucrose for 48 h at 4 °C, frozen in 2-methylbutane between −30 °C and −40 °C and stored at −80 °C. Brains were sectioned using a freezing microtome, and free-floating 20 μm sections were sequentially collected.

For stereology, sections containing the SN were immunostained against TH by immunohistochemistry. Brain sections were first quenched for 5 min in 3% H_2_O_2_-10% methanol (*v*/*v*). Sections were washed three times in 0.1 M Tris-buffered saline (TBS) between incubation steps. After blocking for 1 h with 5% NGS, sections were incubated with rabbit anti-TH antibody (Calbiochem #657012; 1:2,000) at 4 °C for 48 h in TBS + 2% NGS with agitation, then with biotinylated anti-rabbit antibody in TBS + 2% NGS for 60 min. Sections were visualised by incubation with avidin-biotin-peroxidase complex (Immunopure ABC Peroxidase staining kit, Thermo Fisher Scientific) using diaminobenzidine (DB) as a chromogen. Sections were mounted, Nissl stained and coverslipped with DPX mounting medium. The total number of TH-positive neurons in the SNpc was estimated by stereology according to the optical fractionator method, using a computerised system (SteroInvestigator, MBF Bioscience) coupled to a Zeiss Axio Imager D1 microscope. Every fourth section of the whole SNpc was counted by an investigator blinded to the experimental groups.

For double immunofluorescence, a similar protocol was used without the quenching step. Sections were preincubated in blocking buffer (10% BSA, 3% milk and 0.1% Triton-X100 in PBS) for 30 min. The following primary antibodies were incubated together overnight at 4 °C blocking buffer: mouse anti-TH (Chemicon #MAB 5280; 1:1,000) and rabbit anti-GFP (Torrey-Pines Biolabs #TP401). After washing 3 times with PBS, corresponding secondary antibodies coupled to fluorophores were diluted 1:1000 and incubated simultaneously for 1 h 30 min at room temperature in PBS + 10% BSA + 0.1% Triton X-100. After washing three times with PBS, nuclei were stained with DAPI 1 μg/ml in PBS for 5 min and washed once in PBS. Sections were mounted with Mowiol. Images were acquired using an Olympus FSX100 fluorescence microscope with a DP72 incorporated camera and FSXBSW visualisation software (Olympus, Germany).

### In situ PLA

LUHMES cells were differentiated in monolayers onto glass coverslips. Following 6 days of differentiation, cells were fixed with 4% paraformaldehyde for 20 min at room temperature, washed with 0.1 M Gly (pH 7.1), permeabilised with 0.25% Triton X-100 in PBS for 10 min and washed three times with PBS. The interaction between endogenous TRIM41 and endogenous ZSCAN21 was detected using the Duolink® In Situ kit (Olink® Bioscience, Uppsala, Sweden), according to the manufacturer’s instructions, as described previously [41]. Briefly, cells were successively incubated (i) with Duolink® blocking solution for 60 min at 37 °C; (ii) with primary antibodies against TRIM41 (1:250) and ZSCAN21 (clone OTI2E5, 1:50) diluted in the Duolink® antibody diluent overnight at 4 °C; and (iii) with secondary antibodies conjugated with oligonucleotides (PLA probe anti-mouse MINUS and PLA probe anti-rabbit PLUS diluted 1:5 in Duolink® antibody diluent) for 1 h at 37 °C. The cells were washed twice with Duolink® wash buffer A and then incubated with two connector oligonucleotides together with DNA ligase for 30 min at 37 °C in Duolink® ligation buffer. If the two secondary antibodies are in close proximity, this step allows the connector oligonucleotides to hybridise to the PLA probes and form a circular DNA strand after ligation. The cells were washed twice with Duolink® wash buffer A and then incubated, for 100 min at 37 °C, with DNA polymerase in Duolink® amplification buffer, to perform rolling circle amplification (RCA) of the circular DNA, and annealing of the products with fluorescently-labelled complementary oligonucleotides. Cells were washed twice with Duolink® wash buffer B and then once for 5 min with 1 μg/ml DAPI in PBS to stain the nuclei. Coverslips were finally mounted in Mowiol on glass slides. Cells were analysed using a Zeiss LSM980 confocal microscope, with a Zeiss PLAN-APO 63×/1.4 oil immersion objective. Images were acquired using the Zeiss ZEN Blue software. Images were processed using Image J. The number of dots per cell was estimated in one slice, in around 250 cells in each condition, with an automated procedure using plugins (Feature J Laplacian and find maxima) from the Fiji software in a blinded manner.

### In vitro SUMOylation assay

The expression vector pCI-3 × Flag-ZSCAN21 was previously described [20]. The ZSCAN21 K27R and E29A mutants were obtained by site-directed mutagenesis. ZSCAN21 and its mutants were first transcribed and translated in vitro. For this, 1 μg of pCI-3 × Flag-ZSCAN21 was incubated for 1 h at 30 °C in 50 μl of the TNT® T7 coupled wheat germ extract system (Promega, #L5030), according to the instructions of the manufacturer. Equivalent amounts of the different forms of ZSCAN21 (3–5 μl of the in vitro translation reaction) were incubated for 2 h at 37 °C in the presence of 3 μg recombinant SUMO-1 or SUMO-2, 150 ng recombinant His-tagged Aos1/Uba2 (E1 enzyme), 100 ng recombinant Ubc9 (E2 enzyme) and 300 ng recombinant GST-PIASxα (E3 enzyme) in 20 μl shift-assay buffer (20 mM Hepes [pH 7.3], 110 mM KOAc, 2 mM Mg(OAc)_2_, 0.5 mM EGTA, 1 mM DTT, 0.05% Tween 20, 0.2 mg/ml ovalbumin, 1 μg/ml leupeptin, 1 μg/ml aprotinin, 1 μg/ml pepstatin) supplemented with 1 mM ATP. Recombinant proteins used in this assay were produced and purified as previously described [72]. Reaction products were analysed by western blot using an anti-Flag antibody.

### Statistical analysis

The values were shown as mean ± standard error of the mean (SEM) of independent experiments. For qPCR data, the averages of technical triplicates were used for each independent experiment. Data plotting and statistical analyses were performed using GraphPad Prism version 7.0c or 10.4.0 for Mac OS X (GraphPad Software, San Diego, California USA, www.graphpad.com). When comparing two groups, significant differences (^*^P < 0.05, ^**^P < 0.01, ^***^P < 0.001, ^****^P < 0.0001) were determined by a two-tailed unpaired Student’s *t*-test. When comparing more than two groups, significant differences were determined by one-way or two-way analysis of variance (ANOVA), followed by an adequate multi-comparison post-test as indicated in the legends of the figures. Post-tests were performed only when the ANOVA was significant. Normal distribution with similar variance between groups was assumed because n was too small for normality tests. None of the samples were excluded except when a technical problem occurred and, in the case of mice, when no shRNA-associated GFP expression was detected in the SN.

### Supplementary information

The online version contains supplemental material. In particular, supplemental Tables indicate the sequences of siRNAs, shRNAs, CRISPR-Cas9 guide RNA and oligonucleotide template, as well as primers for quantitative PCR and site-directed mutagenesis used in this study. Uncropped original western blots used in the manuscript are also provided as supplementary information.

## Supplementary information

Supplementary text and figures

original Western blots

## Data Availability

All unique materials generated in this study are available from the corresponding author on reasonable request.
