# Supplementary material for: ZSCAN21 mediates the pathogenic transcriptional induction of α-synuclein in cellular and animal models of Parkinson’s disease
Source: Cell Death Dis. 2025 May 16;16(1):394. doi: 10.1038/s41419-025-07722-w (PMC12084422; doi:10.1038/s41419-025-07722-w)
Supplement: Supplementary file 1 — Supplementary text and figures [file 41419_2025_7722_MOESM1_ESM.pdf]

Supplementary text and figures to:

ZSCAN21 mediates the transcriptional induction of α-synuclein in models of Parkinson's disease

By Alina Kozoriz, Stéphan Mora, Maria-Alessandra Damiano, Iria Carballo-Carbajal, Annabelle Parent, Lorena Kumarasinghe, Miquel Vila, Iréna Lassot and Solange Desagher

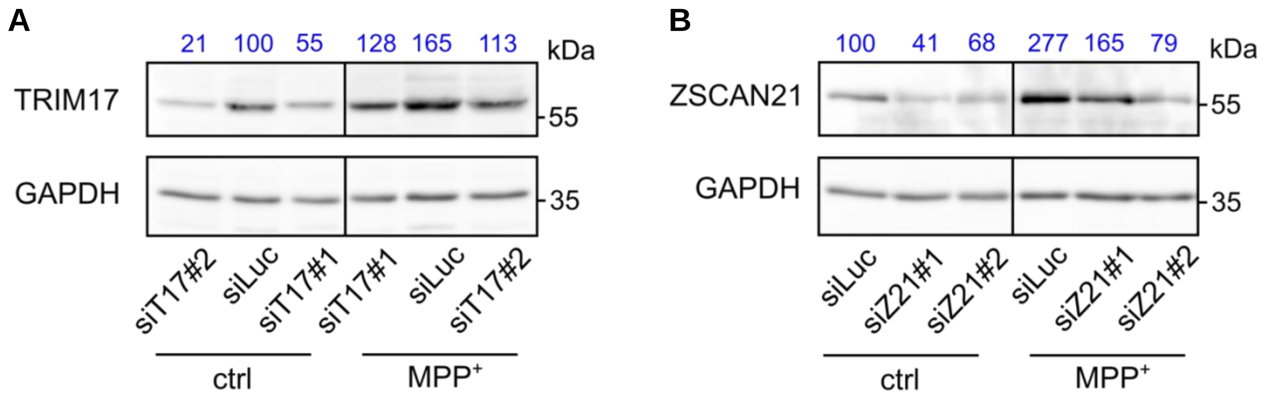

Figure S1: Knock-down of TRIM17 and ZSCAN21 in LUHMES cells. LUHMES cells were transfected with a control siRNA against Luciferase (siLuc), two different siRNAs against TRIM17 (siT17#1 and siT17#2) or two different siRNAs against ZSCAN21 (siZ21#1 and siZ21#2) at the time of plating for 3D-differentiation. After 6 days of differentiation, LUMHES spheroids were treated or not with 2 μM MPP^{+} for 24 h and total proteins were analysed by western blot using antibodies against GAPDH and TRIM17 (**A**) or ZSCAN21 (**B**). The intensity of the TRIM17 or ZSCAN21 bands was quantified, normalized by the intensity of the GAPDH bands and expressed relative to the values obtained with non-treated cells transfected with the control siRNA (siLuc): values in blue above the corresponding bands. For each protein, the two parts separated by a vertical line are from the same gel with the same exposure. These data are representative of two independent experiments.

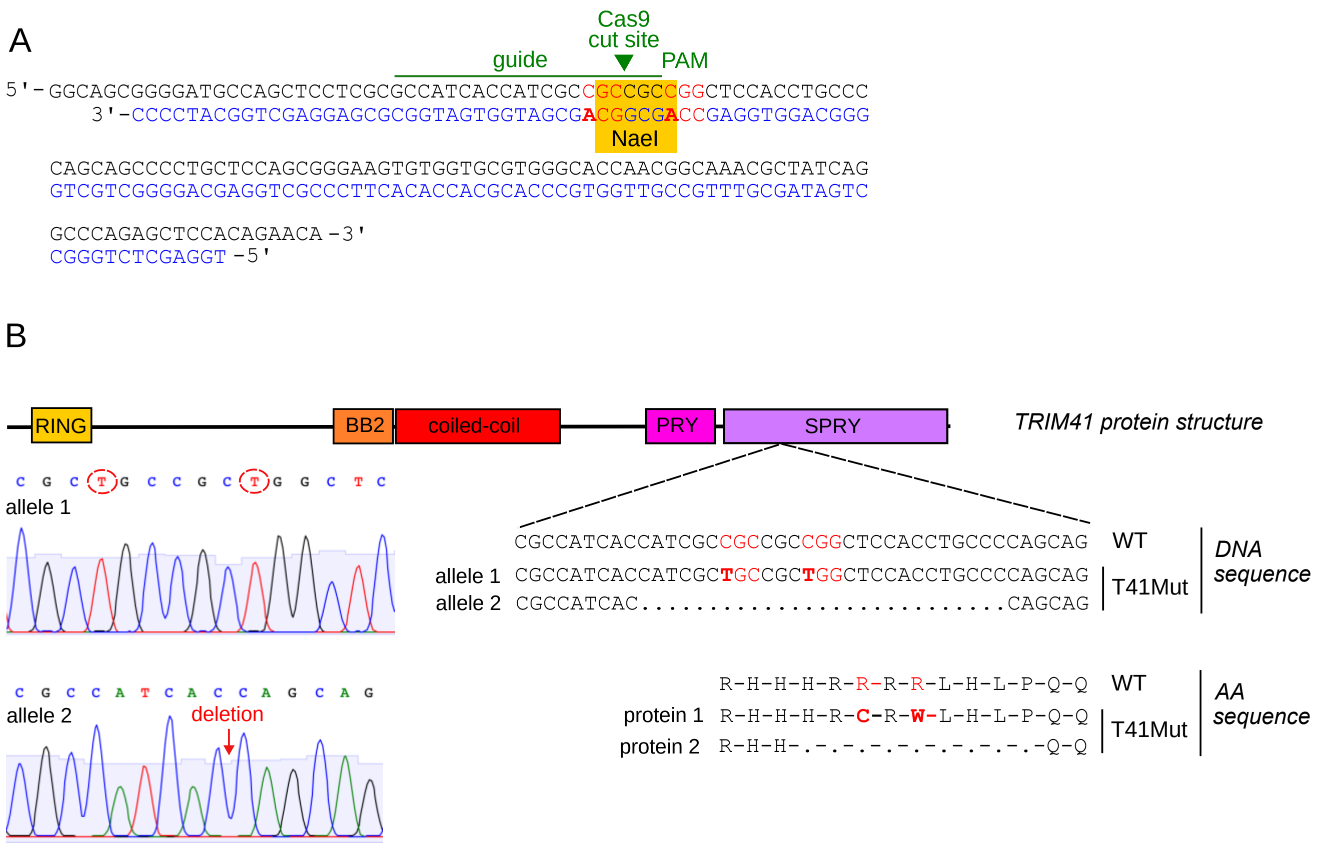

Figure S2: CRISPR-Cas9 genome editing of *TRIM41* in LUHMES cells. A. Genomic DNA sequence of the targeted locus in human *TRIM41* with the binding site of the guide RNA, Cas9 cleavage site and the PAM in green. The codons corresponding to R534 and R536 are in red. The sequence of the donor oligonucleotide is represented in blue, with the nucleotides modified in order to introduce the desired point mutations in genomic DNA in bold. The yellow box underlines the NaeI restriction site that is disrupted if the point mutations are introduced or if small insertions/deletions occur after Cas9 cleavage. Note that introducing the desired mutations disrupts the PAM, preventing further cleavage by Cas9 following DNA repair. **B.** Schematic representations of the different domains of the TRIM41 protein (BB2: B-Box2) indicating the location of the amino acid substitutions. Chromatograms of DNA sequencing for the two alleles of the clone T41Mut are shown, with the two-point mutations in red circles and the location of the deletion indicated with an arrow. The DNA sequences of WT TRIM41 and the two alleles of clone T41Mut are compared, as well as the amino acid sequences of the protein products.

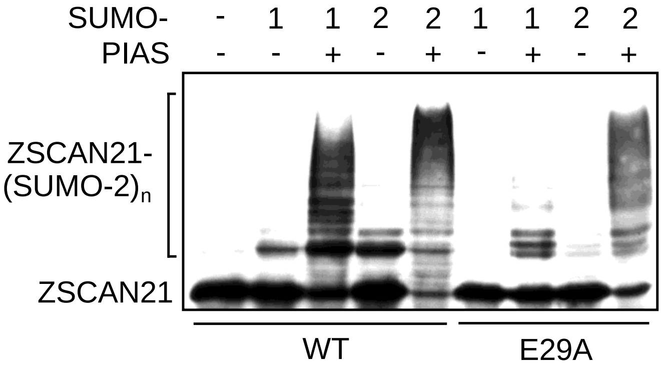

Figure S3: ZSCAN21 is SUMOylated on K27 consensus site. *In vitro* translated 3×Flag-ZSCAN21, either the WT form or the E29A mutant, was incubated with *in vitro* SUMOylation reaction mix (containing E1 and E2 enzymes), in the presence or the absence of SUMO-1 or SUMO-2, in the presence or the absence of the E3 enzyme PIASxα, as indicated, for 1h at 37 °C. Multi-SUMOylated forms of ZSCAN21 were detected by immunoblotting using anti-Flag antibody.

Supplementary Table 1: list of sequences targeted by siRNAs and shRNAs

siRNAs	cDNA targeted sequences (5’ – 3’)
siRNA-Luc	CGTACGCGGAATACTTCGA
siRNA-TRIM17#1	CCAAGTACTTATCCACCTT
siRNA-TRIM17#2	GGTATACTGACAGATGCTT
siRNA-TRIM41	AAGGAGACTTTCAATAGGTGT
siRNA-ZSCAN21#1	CTTAGAGAGGCAGTGCGTAAA
siRNA-ZSCAN21#2	GATCCAAGAAAGGTCCGAGAT
**shRNAs**	**cDNA targeted sequences (5’ – 3’)**
shRNA-Luc	CGCTGAGTACTTCGAAATGTC
shRNA-eGFP	TACAACAGCCACAACGTCTAT
shRNA-Trim17	CTGTTACCCAATTCCACTCTA
shRNA-Zscan21	GCCAGCCCTAAATATGAGTTT

Supplementary Table 2: list of the primers used for quantitative RT-PCR

cDNA	Forward (5’ - 3’)	Reverse (5’ - 3’)
human SNCA pre-mRNA	GCAGAAGCAGCAGGAAAGAC	CCTACCTACACATACCTCTGAC
human SNCA	GCAGAAGCAGCAGGAAAGAC	ACCACTGCTCCTCCAACATT
human TRIM17 pre-mRNA	AGGACATGGAGTACCTTCGG	ACAGACGTGGAAATGCAGGA
human TRIM17	GACATGGAGTACCTTCGGGA	GCAGTCTCCTCTTCTTCCGT
human TRIM41	AGCTCTTCTGCGAGGTAGAC	CTCTTCAGCCAGAAACCGTG
human ZSCAN21	CAAATACGAGTCTTGGGGGC	TCTAAGCTGGCCTCAGGTTT
human B2M	GCGCTACTCTCTCTTTCTGG	AGAAAGACCAGTCCTTGCTGA
human GAPDH	CCATCTTCCAGGAGCGAGAT	GGTTCACACCCATGACGAAC
human GUSB	TGGGTCTGGATCAAAAACGC	TTGTCTCTGCCGAGTGAAGA
human HMBS	ACACACAGCCTACTTTCCAAG	AGAATCTTGTCCCCTGTGGT
human HPRT1	TGACACTGGCAAAACAATGCA	GGTCCTTTTCACCAGCAAGCT
human RPL31	ACGAAGTGGTAACCCGAGAA	TCCCATCTCCTTCATGGCAA

human RSP16	GGTCTCATCAAGGTGAACGG	CACGGATGTCTACACCAGCA
human SDHA	TGGGAACAAGAGGGCATCTG	CCACCACTGCATCAAATTCATG
human TBP	GTTCTGGGATTGTACCGCAG	TTATATTCGGCGTTTCGGGC
human YWHAZ	GTCATCTTGGAGGGTCGTCT	GCTTGTGAAGCATTGGGGAT
mouse B2M	TATGCTATCCAGAAAACCCCTCAA	GTATGTTCGGCTTCCCATTCTC
mouse Hmbs	ACTCTGCTTCGCTGCATTG	AGTTGCCCATCTTTCATCACTG
mouse Trim17	ACTGAGTGGCAGGAGAGAGTGAA	CCAGAAACAAGACCACCTTCTGA
mouse Zscan21	GACTGGTGAGGAGGAGGTTT	ATCATGGGGATGACGGTTCT

Supplementary Table 3: list of the primers used for ChIP analysis by quantitative PCR

**DNA**	**Forward (5' - 3')**	**Reverse (5' - 3')**
SNCA promoter	GCTTGTTCATGAGTGATGGGT	CCTCTGACTCAGTCCACCTTT
SNCA "enhancer 1"	GCCCCGGTGTTATCTCATTC	CCTCTCCAATTACGATTTTAGCAC
SNCA "enhancer 2"	GTGCAGCGGTAGTTTAGCTT	TTTCCTGGATGCTCAGTGGT
SNCA "enhancer 4"	CTGCTTGGTTTTCCCTTTTG	CTGCAAACGCTTCTGTTTTG
SNCA "enhancer 7"	TATATTCTCCAAATTTCCTGTCCTTCC	TAAGGTTCCTCTTGAATCACAATTTATC

Supplementary Table 4: targeted sequence, DNA template and primers for CRISPR Cas9 genome editing of TRIM41

**DNA**	**Forward (5' - 3')**	**Reverse (5' - 3')**
Primers to amplify the genomic DNA around Cas9 cut site	GAGGTTGCTGACCATCCCAA	GACCAGGCAGCTGTTCTCAT
Sequencing primer	ACTACTGGGAGGTAGAGGTG	
sequence targeted by the crRNA (PAM in red)	GCCATCACCATCGCCGCCGCCGG	
DNA template (targeted codons in red, desired point mutations in bold, *: phosphorothioates)	T*G*GAGCTCTGGGCCTGATAGCGTTTGCCGTTGGTGCCCACGCACCACACTTCCCGCTGGAGCAGGGGCTGCTGGGGCAGGTGGAGCC**A**GCGGC**A**GCGATGGTGATGGCGCGAGGAGCTGGCATCC*C*C	

Supplementary Table 5: PCR primer pairs for directed mutagenesis of ZSCAN21
(modified codons in red, mutations in bold)

DNA	Forward (5' - 3')	Reverse (5' - 3')
ZSCAN21 K27R	GCCTCTGATGGTAA**G**AGTCGAG GAG	CTCCTCGACT**C**TTACCATCAGAGGC
ZSCAN21 E29A	GATGGTAAAAGTCG**C**GGAGAAA GAAGAG	CTCTTCTTTCTCC**G**CGACTTTTACCATC
