# Supplementary material for: ZSCAN21 mediates the pathogenic transcriptional induction of α-synuclein in cellular and animal models of Parkinson’s disease
Source: Cell Death Dis. 2025 May 16;16(1):394. doi: 10.1038/s41419-025-07722-w (PMC12084422; doi:10.1038/s41419-025-07722-w)

Uncropped western blots

Revised figure 1E

anti-GAPDH

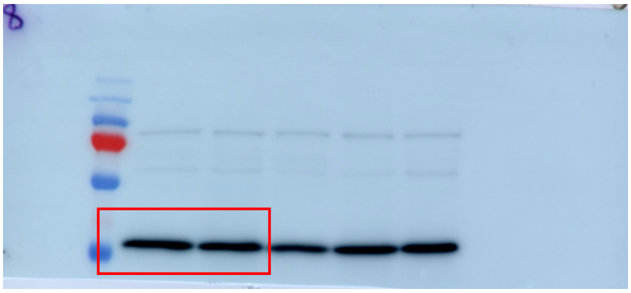

anti-P-ser129-α-synuclein

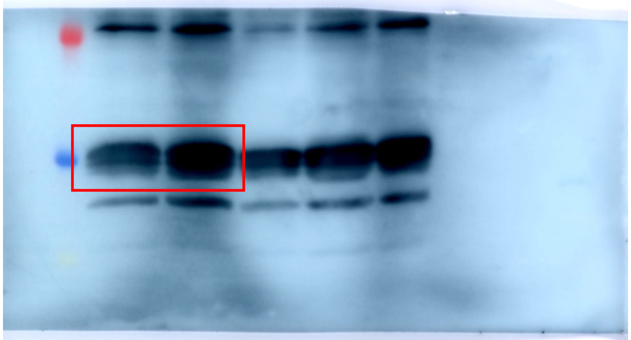

anti-α-synuclein

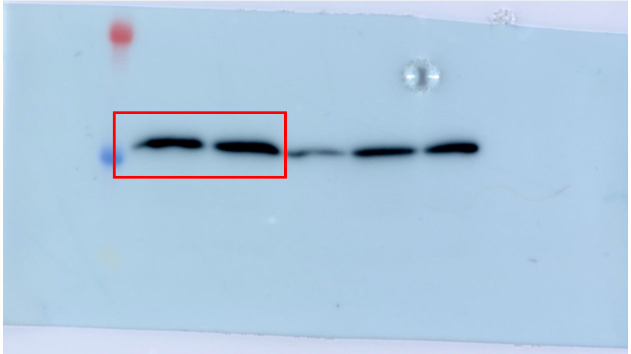

Revised figure 1F

Triton X-100 fractions

anti-GAPDH

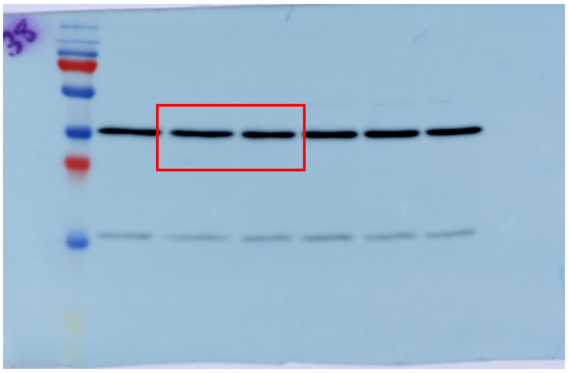

anti-α-synuclein

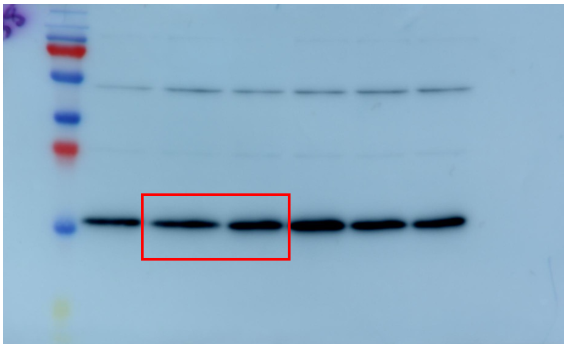

SDS fractions

anti-α-synuclein

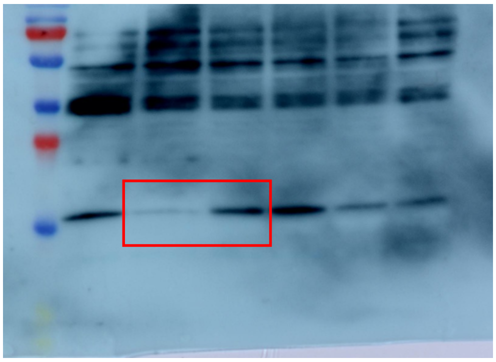

amidoblack staining

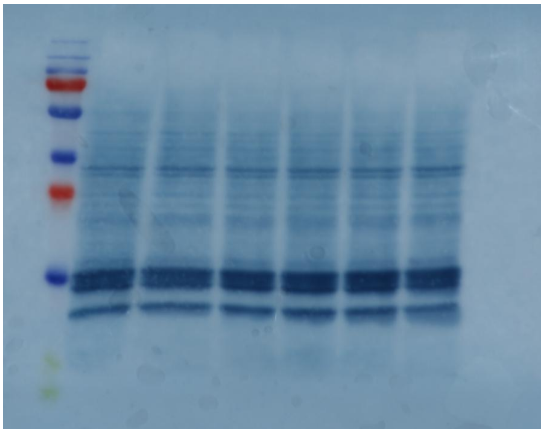

Revised figure 2B

anti-TRIM41

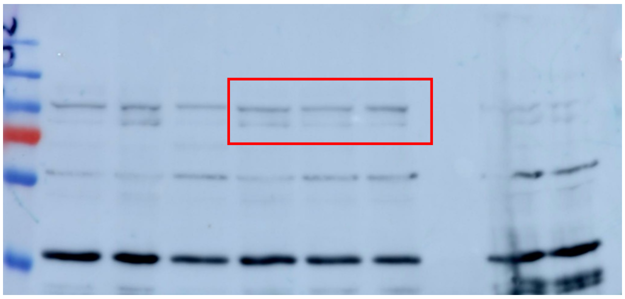

anti-TRIM17

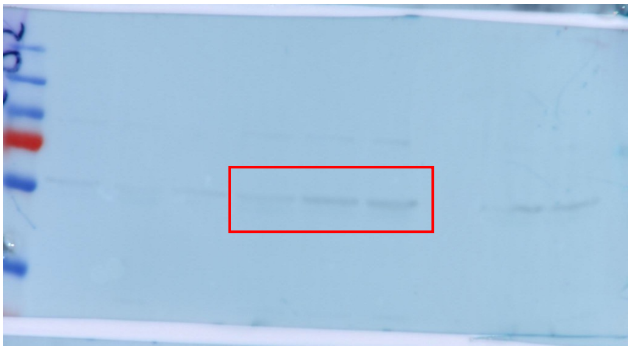

anti-GAPDH

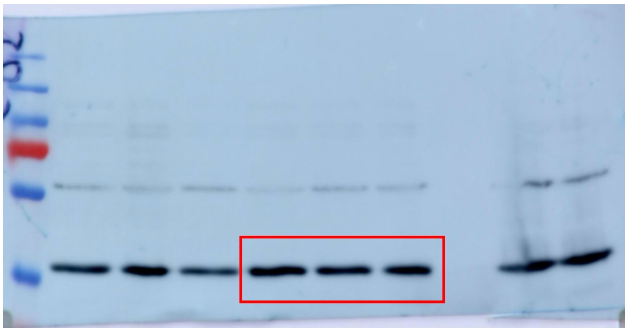

Revised Figure 2C

anti-ZSCAN21

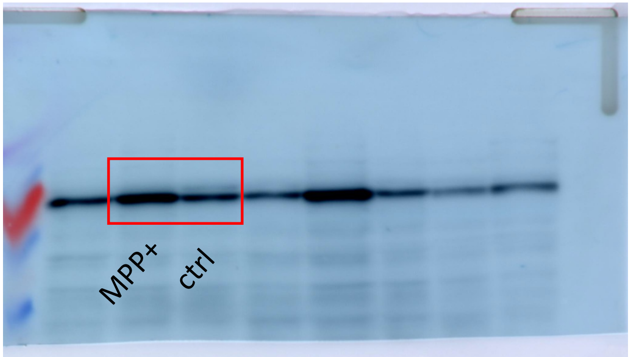

anti-GAPDH

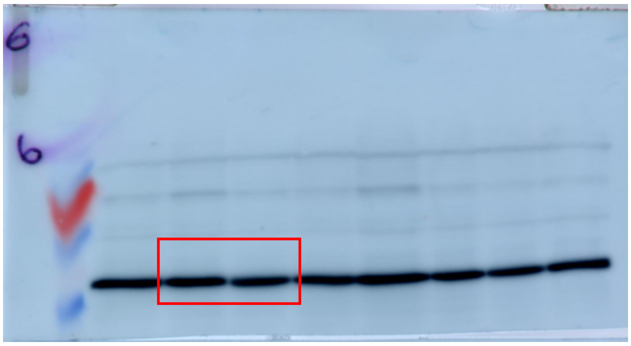

Revised figure 2E

control conditions

anti-ZSCAN21

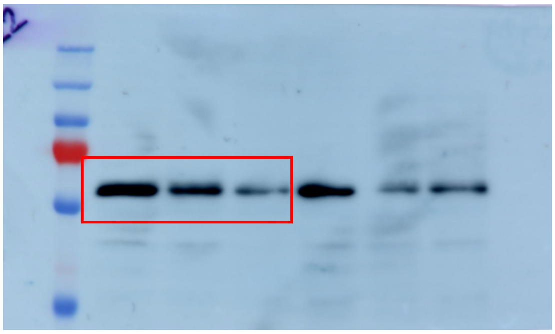

anti-GAPDH

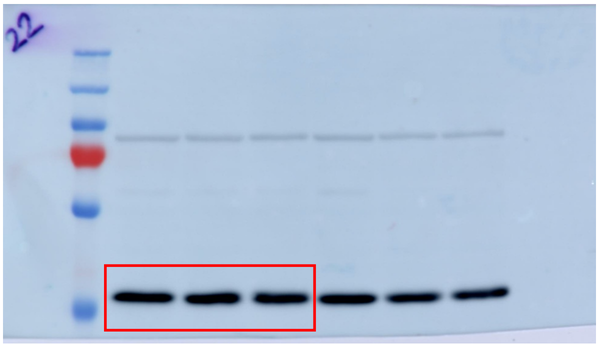

MPP^+ conditions

anti-ZSCAN21

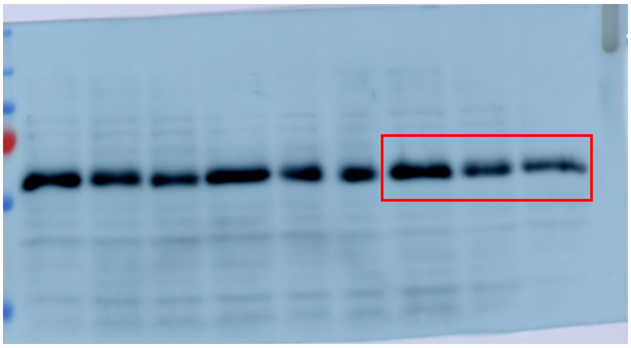

anti-GAPDH

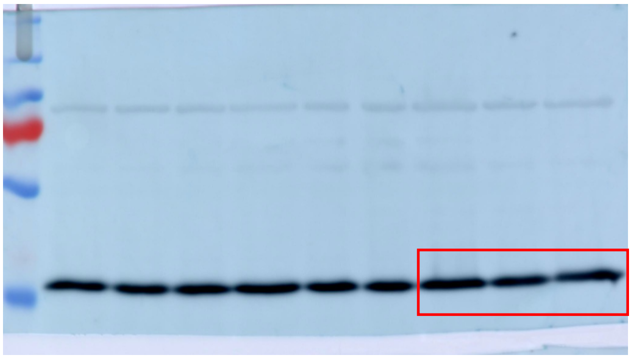

Revised figure S1

anti-TRIM17

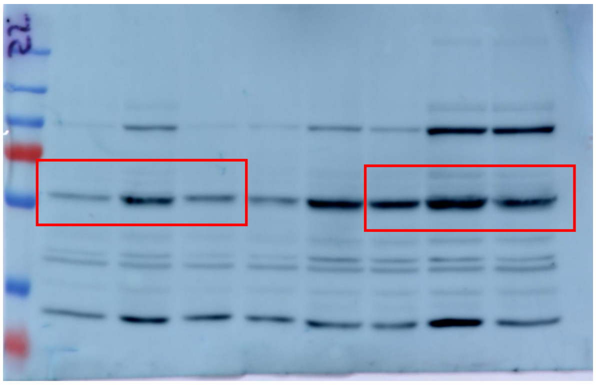

anti-ZSCAN21

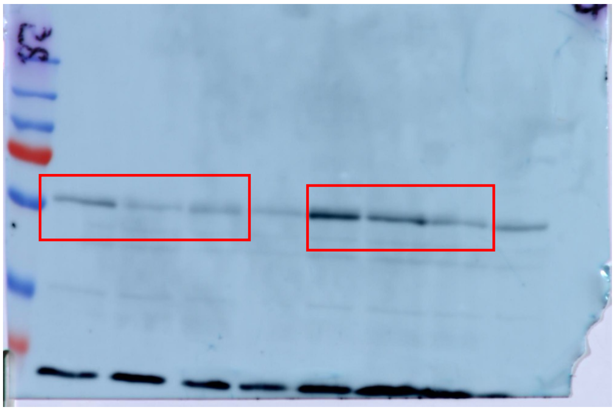

anti-GAPDH

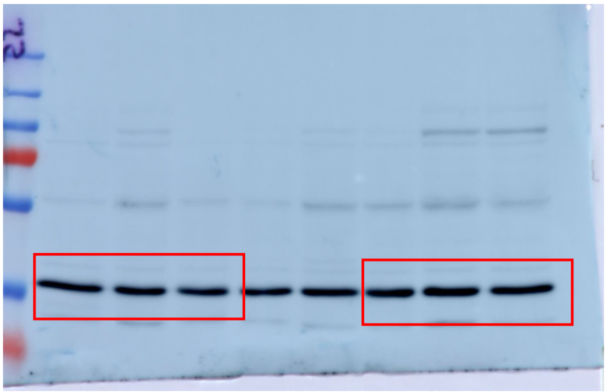

anti-GAPDH

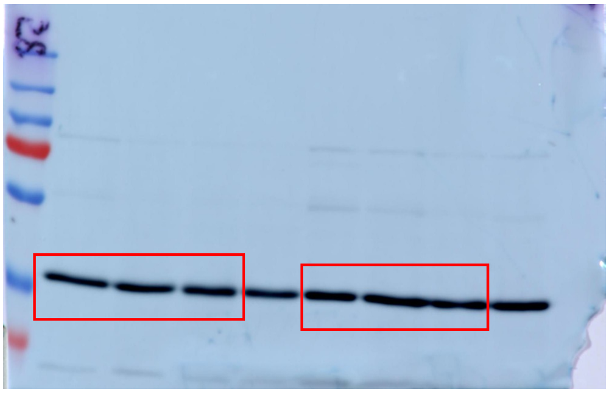

Revised Figure 6B

anti-ZSCAN21 for WT

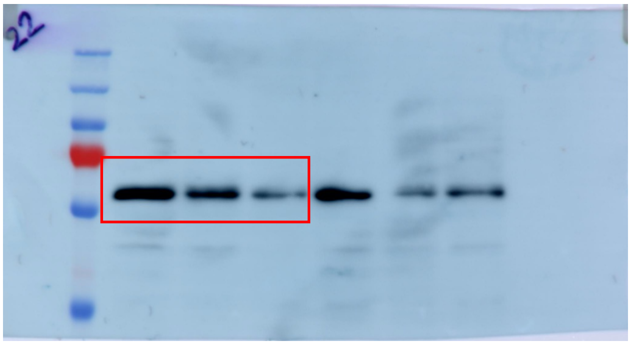

anti-GAPDH for WT

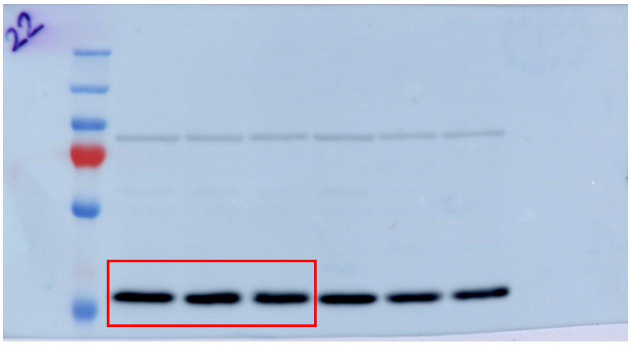

anti-ZSCAN21 for T41Mut

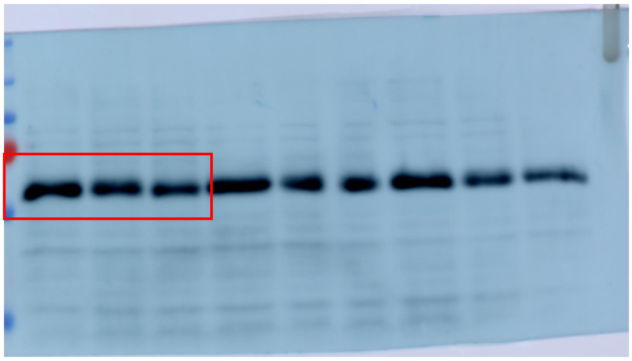

anti-GAPDH for T41Mut

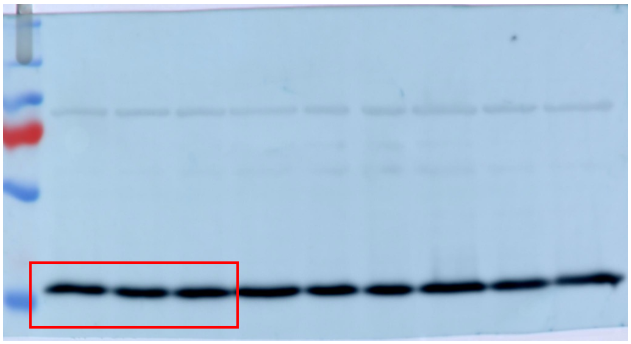

Revised Figure 6C

anti-ZSCAN21

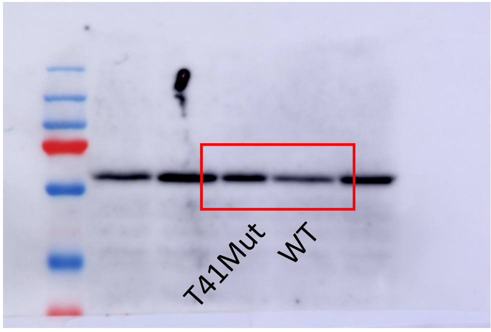

anti-actin

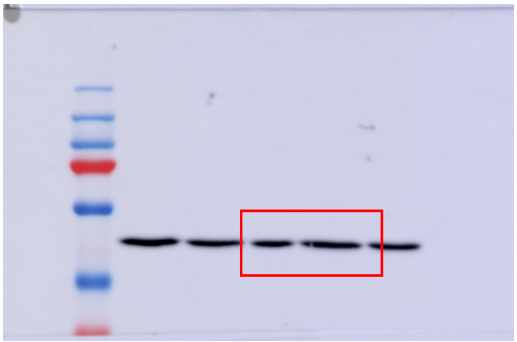

Revised Figure 6E

anti-GAPDH

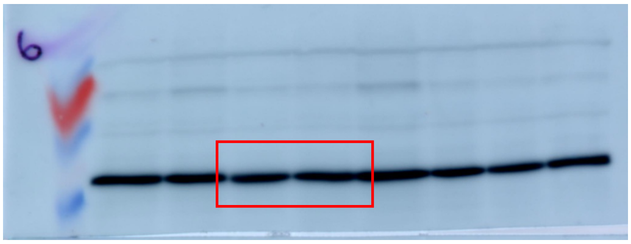

anti-α-synuclein

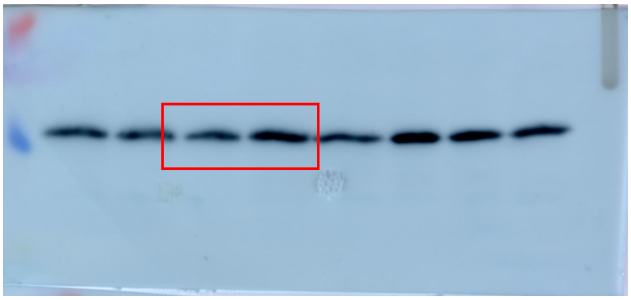

Revised figure 7B

anti-Flag

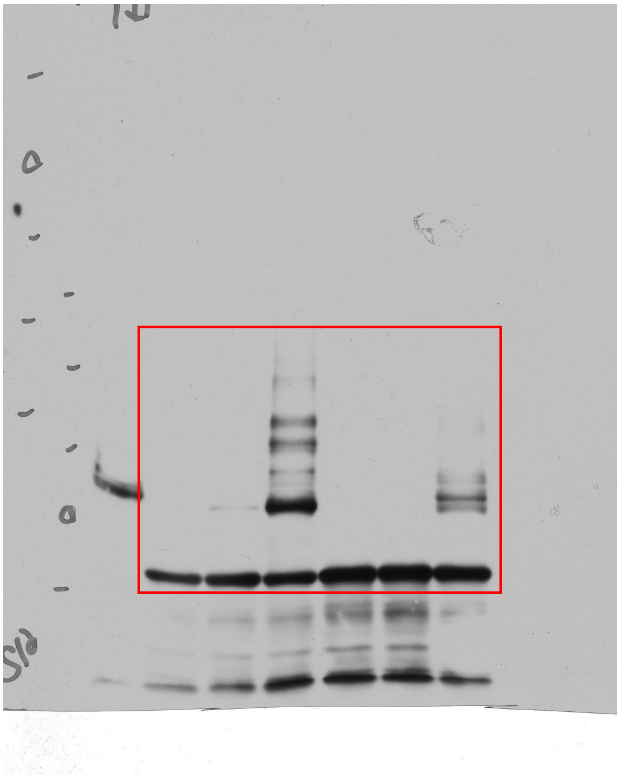

Revised figure S3

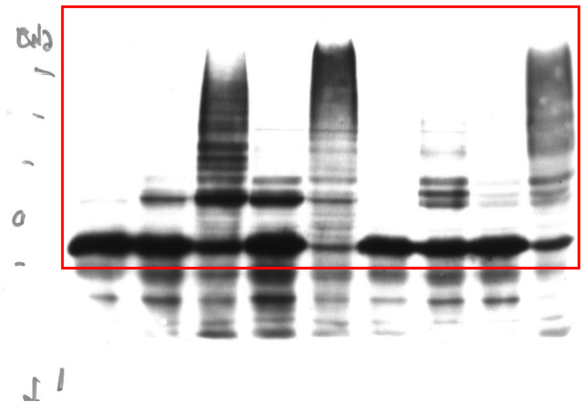

Revised figure 7D

anti-ZSCAN21 for ctrl

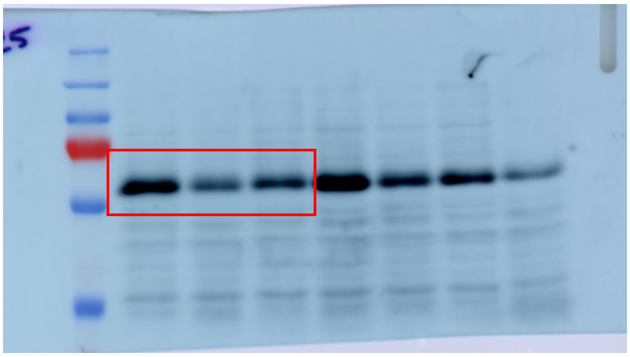

anti-GAPDH for ctrl

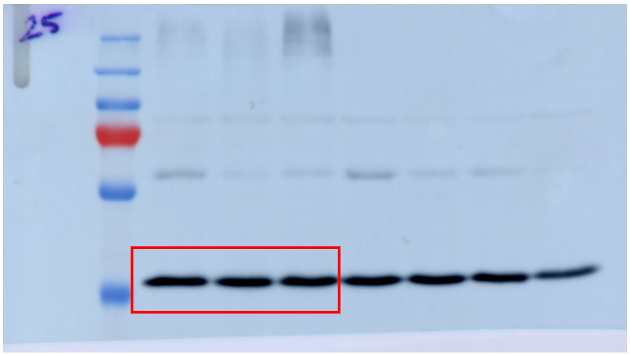

anti-ZSCAN21 for TAK-981

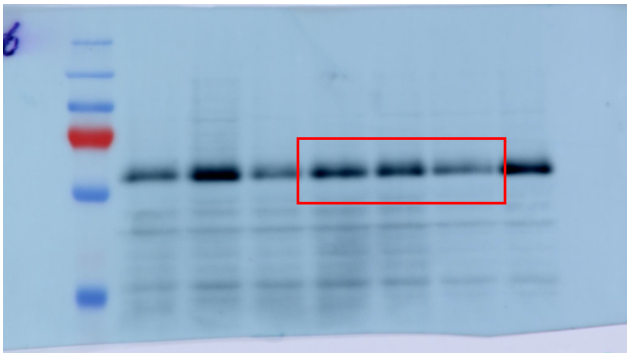

anti-GAPDH for TAK-981

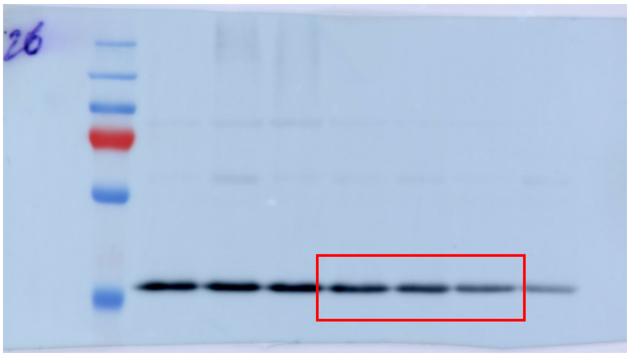

Revised figure 7E

anti-ZSCAN21

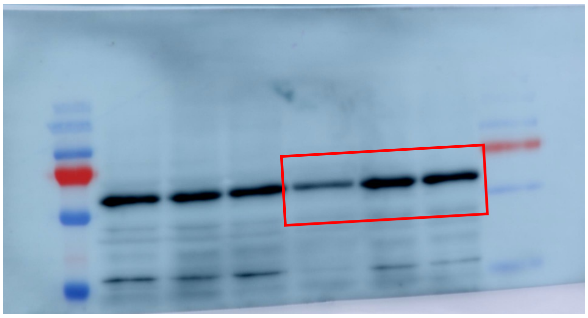

anti-SUMO-2

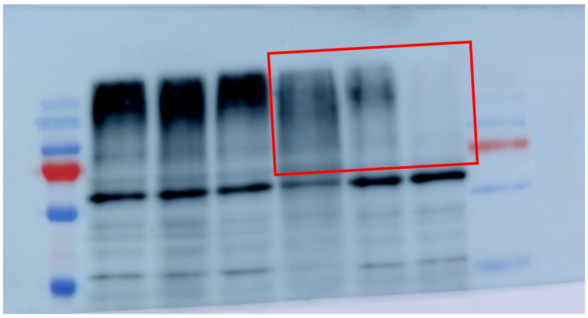

anti-GAPDH

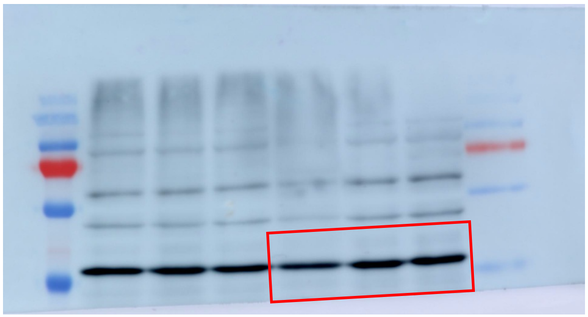

anti-α-synuclein

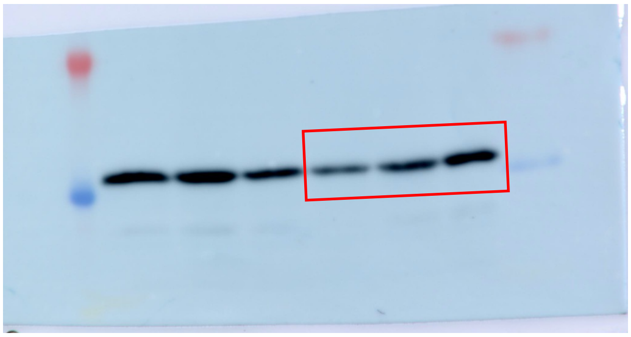

Supplement: Supplementary file 2 — original Western blots [file 41419_2025_7722_MOESM2_ESM.pdf]
